# A Short Sequence Targets Transmembrane Proteins to Primary Cilia

**DOI:** 10.3390/cells13131156

**Published:** 2024-07-06

**Authors:** Viviana Macarelli, Edward C. Harding, David C. Gershlick, Florian T. Merkle

**Affiliations:** 1 Institute of Metabolic Science, University of Cambridge, Cambridge CB2 0QQ, UK; vm412@cam.ac.uk (V.M.); ech66@cam.ac.uk (E.C.H.); 2 Cambridge Stem Cell Institute, University of Cambridge, Cambridge CB2 0AW, UK; 3 Cambridge Institute for Medical Research, University of Cambridge, Cambridge CB2 0XY, UK; dg553@cam.ac.uk

**Keywords:** primary cilia, targeting sequence, protein trafficking, iPSC, pluripotent

## Abstract

Primary cilia are finger-like sensory organelles that extend from the bodies of most cell types and have a distinct lipid and protein composition from the plasma membrane. This partitioning is maintained by a diffusion barrier that restricts the entry of non-ciliary proteins, and allows the selective entry of proteins harboring a ciliary targeting sequence (CTS). However, CTSs are not stereotyped and previously reported sequences are insufficient to drive efficient ciliary localisation across diverse cell types. Here, we describe a short peptide sequence that efficiently targets transmembrane proteins to primary cilia in all tested cell types, including human neurons. We generate human-induced pluripotent stem cell (hiPSC) lines stably expressing a transmembrane construct bearing an extracellular HaloTag and intracellular fluorescent protein, which enables the bright, specific labeling of primary cilia in neurons and other cell types to facilitate studies of cilia in health and disease. We demonstrate the utility of this resource by developing an image analysis pipeline for the automated measurement of primary cilia to detect changes in their length associated with altered signaling or disease state.

## 1. Introduction

Primary cilia are non-motile structures that protrude from the plasma membrane of most eukaryotic cell types. While their exact structure and protein composition partly varies by cell type, it is widely accepted that they play key sensory roles [1]. For example, photoreceptor outer segments are a type of specialized primary cilia, olfactory sensory neurons utilize primary cilia to efficiently detect odorants, and primary cilia in hypothalamic neurons likely sense neuropeptides critical for normal body weight regulation [2,3,4]. This sensory specialization is mediated by the ciliary localisation and concentration of certain ion channels, receptor tyrosine kinases, G-protein-coupled receptors (GPCRs), and their downstream mediators [1]. The small ciliary volume allows these proteins to be in close proximity and facilitates dramatic changes in the concentration of second messengers such as cAMP or Ca^2+^ [5]. The importance of ciliary signaling is further exemplified by the range of diseases that result from ciliary dysfunction.

Selective ciliary protein localisation is regulated by the distinct structural features of primary cilia, which are organized around a microtubule-based structure known as the axoneme. Ciliary proteins and cargo vesicles are actively transported up and down the axoneme by kinesin and dynein molecular motors, respectively, using intraflagellar transport complexes A (IFT-A) and B (IFT-B). The IFT machinery assembles into larger macromolecular structures known as ‘IFT trains’ that can be observed along the axoneme or at the ciliary base awaiting entry into the cilium [6,7]. In order to reach the cilium, after being synthesized in the cytoplasm, many ciliary proteins harbor a ciliary targeting or localisation sequence (CTS) which is recognized by complexes of proteins at the base of the cilium [8]. In contrast to signal peptides that determine canonical translocation with well-characterized sequences, CTS sequences are diverse and their interaction partners and pathways for ciliary targeting are not well understood [9,10]. Furthermore, some membrane proteins utilize non-traditional pathways in their trafficking to the cilium by bypassing the Golgi altogether [11].

Once at the cilium, ciliary entry is regulated by the transition zone (TZ) at the proximal end of the axoneme near its connection to the basal body, where it is anchored to the ciliary membrane via Y-link protein complexes. The TZ forms a selectively permeable diffusion barrier for both proteins and lipids, effectively separating the ciliary membrane from the rest of the cell [12]. The ciliary entry of proteins > 50–100 kDa and vesicles containing membrane-associated proteins is regulated by protein complexes named after ciliopathies resulting from their functional disruption [5,12,13,14,15].

Experimentally targeting proteins to primary cilia is a powerful way to study ciliary structure and function in live cells to characterize the ciliary proteome, revealing therapeutic targets that act in primary cilia, or better understand the function of cilia in health and disease. Others have shown that fluorescent reporters localize to primary cilia when fused to the coding sequences of somatostatin receptor 3 (SSTR3) [16,17,18], the serotonin receptor 6 (5HT6), or ADP ribosylation factor-like GTPase 13B (ARL13B) [19,20]. The same is true for the first 203 residues of nephronophthisis 3 (NPHP3), which contains an N-terminal myristoylation site and an N-terminal coiled-coil domain required for proper ciliary targeting [21] and is sufficient to target fusion proteins to primary cilia in some cell types [22]. However, existing ciliary targeting constructs such as N-terminal NPHP3-GFP do not localize to cilia in some cells derived from human-induced pluripotent stem cells (hiPSCs), limiting opportunities to study cilia in diverse human cell types. In addition, even in cases where the off-target expression of these constructs is not an issue, overexpressing whole functional proteins or protein domains may inadvertently alter normal ciliary function, making interpretation challenging. An ideal CTS would facilitate specific ciliary localisation in many cell types, be compact in order to preserve protein function, and not perturb the endogenous function of the primary cilium.

We set out to find new methods to specifically label the primary cilia of human iPSC-derived neurons, including cortical neurons and hypothalamic neurons reported to be enriched in obesity-associated GPCRs, such as the melanocortin 4 receptor (MC4R) [2,23]. We therefore generated cytoplasmic or membrane-localized reporter constructs and tested the ability of candidate CTSs to mediate ciliary targeting. We identified two sets of CTSs, one from the Melanin-concentrating hormone receptor 1 (MCHR1) and one from polycystin 2 (PC2/PKD2), sufficient to target a transmembrane construct to primary cilia in hPSC-derived neurons and all three other commonly used ciliated cell lines that we tested. We therefore generated stable iPSC lines expressing a construct with or without the 3x CTS array, which labels either the primary cilium or plasma membrane, respectively, in hiPSC-derived cell types. We demonstrate the utility of this tool by testing the effect of compounds that alter ciliary length in live human neurons, and provide the reagents we established as a resource to the community.

## 2. Materials and Methods

### 2.1. Cell Culture

All cultured cells in this study were maintained at 37 °C in humidified incubators at 5% CO_2_ and 20% O_2_. Human-induced pluripotent stem cell (hiPSC) lines KOLF2.1J (P24 to P38) [24] and WTC-11 (P40 to P45) [25] were maintained as previously described [26]. Briefly, cells were maintained in StemFlex medium on plates coated with Geltrex, and passaged with 0.5 mM EDTA (Ethylenediaminetetraacetic acid) when 70–80% confluent, and replated in the presence of 10 µg/mL Rock Inhibitor Y-27632. The IMCD3 cell line (P20–30) [27] was maintained in DMEM-F12 medium supplemented with 2.5 mM L-Glutamine, 10% fetal bovine serum (FBS), and 1% Penicillin-Streptomycin. NIH-3T3 (3T3) fibroblasts (P5 to P10) were maintained in DMEM medium supplemented with 10% FBS, while RPE1-hTERT cells (P7 to P12) were maintained in DMEM-F12 medium supplemented with 2.5 mM L-Glutamine, 10% FBS, and 1% Penicillin-Streptomycin. IMCD3, 3T3, and RPE1 cells were passaged with Trypsin—EDTA solution when 70–80% confluent.

### 2.2. Cortical Neuron Differentiation

Cortical neurons were generated by inducibly expressing the transcription factor neurogenin-2 (NGN2) with doxycycline as previously described [28] and detailed below. The WTC-11-NGN2-dCAS9 hiPSC line, in which NGN2 transgene was stably integrated into the *AAVS1* safe-harbor locus under a doxycycline-inducible promoter and dCAS9 was inserted into the CLYBL safe-harbor locus [29], was a gift from Michael E. Ward. The KOLF2.1J-NGN2 hiPSC line was generated by two consecutive rounds of transfection with a piggyBac transposon vector containing human NGN2 under a dox-inducible promoter [30] together with a plasmid encoding the piggyBac transposase in a 3:1 ratio (vector:transposase) using the P3 Primary cell 4D-Nucleofector™ kit (Lonza, Cambridge (UK)). Prior to targeting, all plasmid DNA was purified by Triton X-114 isothermal extraction as described previously [31]. A total of 3 µg DNA was delivered to 8 × 10^5^ hiPSCs dissociated to a single-cell suspension with TrypLE Express, and cells were plated into 1 well of a 6-well plate in the presence of 10 µg/mL Rock Inhibitor Y-27632. After allowing the cells to recover for 72 h, they were exposed to 5 μg/mL of blasticidin for six days to select for cells that had stably integrated the NGN2 transgene. Finally, twelve clonal lines were isolated, validated by assessing the efficiency of neuronal differentiation, and one was selected to use for experiments.

Human iPSCs were dissociated with 1× TrypLE Express and differentiated for three days in neuronal induction medium (DMEM/F12 with HEPES, N-2 Supplement, Glutamax, MEM non-essential amino acids (NEAA) supplemented with 2 µg/mL Doxycycline, 10 µg/mL Rock Inhibitor Y-27632, and 50 µM 2-Mercaptoethanol) at a density of 1 × 10^5^ cells per cm^2^ on Geltrex-coated plates. Rock Inhibitor was withdrawn after 24 h. After three days, cells had adopted neuronal morphology and were dissociated with a mixture of 1×TrypLE Express and 200 U/mL papain (10:1) and washed with medium supplemented with 2 mg/mL DNAse I (1:60), and replated in cortical medium supplemented with 10 µg/mL Rock Inhibitor Y-27632 at 3 × 10^5^ cells per cm^2^ onto plates coated with 0.1 mg/mL Poly-Ornithine. Cortical medium consisted of BrainPhys medium containing B27 Supplement, 10 ng/mL human neurotrophic factors (hGDNF, hBDNF, and hNT-3) and 1 µg/mL mouse Laminin. For neuronal maintenance, 50% cortical medium without Rock Inhibitor was replaced every 2–3 days until day 10 to 20 of differentiation, when neurons were used for experiments. When differentiating the KOLF2.1J PiggyBac line, 1 µM uridine and 1 µM fluorodeoxyuridine were added to the media from day 2 onward to suppress mitotic cells.

### 2.3. Hypothalamic Neuron Differentiation

Human iPSCs were differentiated to hypothalamic neurons by directed differentiation as previously described [26,32,33]. Briefly, hiPSCs were dissociated with TrypLE Express and plated onto Geltrex-coated plates at a density of 1 × 10^5^ cells/cm^2^ in StemFlex medium supplemented with 10 µg/mL Rock Inhibitor Y-27632. The following day, medium was changed to N2B27 medium (1:1 Neurobasal-A to DMEM/F12 with GlutaMAX media, 1× Glutamax, 0.075% Sodium bicarbonate, 1× MEM NEAA, 200 mM ascorbic acid, 1× penicillin-streptomycin, 1× B27 supplement, and 1× N2 supplement). Small molecule modulators of developmentally important signaling pathways were then sequentially added to induce hypothalamic fate as previously described [26]. On day 13 of differentiation, hypothalamic progenitors were dissociated with a mixture of 1× TrypLE Express and 200 U/mL papain (10:1), washed with medium supplemented with 2 mg/mL DNAse I (1:60), and re-plated at a concentration of 3 × 10^5^ cells/cm^2^ onto 4 μg/mL Laminin 511-coated plates in N2B27 medium (see above) supplemented with 10 μg/mL BDNF and 10 µg/mL Rock Inhibitor Y-27632. The following day, media were changed to Synaptojuice medium 1 (N2B27 medium, 2 μM PD0332991 isethionate, 5 μM DAPT, 370 μM CaCl_2_, 1 μM LM22A4, 2 μM CHIR99021, 300 μM GABA, and 10 μM NKH447) supplemented with 10 μg/mL BDNF. After 1 week, media were switched to Synaptojuice 2 (N2B27 medium, 2 μM PD0332991 isethionate, 370 μM CaCl_2_, 1 μM LM22A4, and 2 μM CHIR99021) and cells were cultured for at least another week. Complete medium change was performed every 2 days and cells were used for experiments from day 30 of differentiation onwards.

### 2.4. Cloning of Fusion Protein Constructs Containing Candidate CTSs

To test the ability of candidate ciliary targeting sequences (CTSs) to localize a fluorescent protein (mScarlet-I) to primary cilia, DNA fragments containing three repetitions of a CTS from known ciliary proteins (Melanin-concentrating hormone receptor 1 (MCHR1), Polycystin 2 (PC2/PKD2), Cystin (CYS1), Somatostatin receptor 3 (SSTR3), Kinesin family member 17 (KIF17), Neuropeptide Y receptor Y2 (NPY2R), and Rhodopsin (RHO) flanked by flexible glycine and serine linkers (GGGGS)) were synthesized as double-stranded DNA by Integrated DNA Technologies and Twist Bioscience. Each fragment was then cloned into the pScarlet-N1 plasmid (RRID:Addgene_128060) from Oskar Laur, previously linearized by digestion with BmtI and PstI, using the In-Fusion HD EcoDry Cloning Plus kit (Cyt-Cilia constructs). The resulting plasmids were verified by restriction digestion and Sanger sequencing, and we refer to those with expected sequences as ‘cytoplasmic’ constructs. The unmodified mScarlet vector served as a negative control.

To generate fusion proteins able to localize to the ciliary membrane, the Interleukin-2 (IL-2) signal peptide, a HaloTag extracellular domain and a transmembrane domain (TM) were synthesized ahead of the 3xCTS array (see above) as double-stranded DNA by Twist Bioscience. The TM domain consisted of 22 Leucine amino acids followed by four positively charged amino acids to stabilize the TM domain in the plasma membrane and aid export through the biosynthetic secretory pathway [34]. A DNA fragment lacking only the CTSs array was also synthesized as a control (TM control). Each fragment was then cloned into the pScarlet-N1 plasmid as described above, and resulting plasmids were verified by restriction digestion and Sanger sequencing. We refer to those with expected sequences as ‘transmembrane’ constructs. A plasmid containing the IL2 signal peptide, HaloTag, TM domain, and mScarlet but lacking a CTS served as a negative ‘TM’ control.

### 2.5. Transient Transfection in Cell Lines and hPSC-Derived Neurons

To test for the ciliary localisation of the candidate plasmids described above or control plasmids including Cilia-APEX, all plasmid DNA was purified by Triton X-114 isothermal extraction as described previously [31] to remove endotoxin and was transiently transfected into different cell types. Cilia-APEX (pEF5B-FRT-cilia-APEX) enabled GFP and APEX to be targeted to cilia in many cell types via the N-terminal 203 residues of NPHP3 and was a gift from Maxence Nachury (RRID:Addgene_73186) [22]. IMCD3, 3T3, and RPE1 cells were transiently transfected with these and our cloned constructs using Lipofectamine™ LTX Reagent (ThermoFisher Scientific, Altrincham (UK)), according to manufacturer’s instructions, when 70–80% confluent. Prior to transfection, the FBS in the media was reduced to 0.2% and cells were then serum-starved for the following 48 h. Cells were then fixed and imaged. Due to the low efficiency of lipofection in human neurons, plasmids were introduced to these cells by electroporation. Specifically, 1 × 10^6^ hiPSC-derived neurons were dissociated with 1x TrypLE Express supplemented with 200 U/mL papain (10:1) and resuspended in 100 μL 1x Opti-MEM I Reduced Serum medium with 2 μg endotoxin-free plasmid DNA. Electroporation was performed using the NEPA21 electroporator (Sonidel,Dublin (Ireland)) (Poring Pulse: 150 V, 5 ms length, 50 ms interval, 2 times; Transfer Pulse: 20 V, 50 ms length, 50 ms interval, 5 times). After electroporation, cells were resuspended in 400 μL of neuronal maturation medium supplemented with 10 μM Rock inhibitor, and plated at density of 6 × 10^5^ cells per cm^2^ in 96-well PhenoPlate microplates. Half medium was changed daily for 72 h, until cells were fixed and imaged.

### 2.6. Generation of Stable hPSC Lines Carrying the Ciliary Targeting Construct

To generate targeting vectors for stable cell line generation, we performed site-directed mutagenesis on pEF5B-FRT-cilia-APEX (RRID:Addgene_73186) to convert APEX to APEX2, and then PCR amplified the coding sequence of APEX2 and cloned it into the Age1 restriction site downstream of mScarlet to produce TM-Scarlet-APEX2, TM-PKD2(3xCTS)-Scarlet-APEX2, and TM-MCHR1(3xCTS)-Scarlet-APEX2 plasmids. These sequences were then PCR amplified, and PCR products were digested with DpnI and cloned into the pENTR™ TOPOR Vector using the pENTR™/D-TOPO™ Cloning Kit (ThermoFisher Scientific, Altrincham (UK)). The resulting pENTR plasmids were then used to efficiently shuttle the constructs into a destination vector using Gateway™ LR Clonase™ II (Invitrogen, Paisley (UK)) to generate the plasmids to target a single copy of each construct to the adeno-associated virus integration, intron 1 (AAVS1) locus of hiSPC lines.

The *AAVS1* destination vector was assembled by PCR amplification and Gibson assembly from the pDTA-TK from Robert Benezra’s group (RRID:Addgene_22677) [35] to introduce negative selection cassettes flanking the *AAVS1* homology arms from the Gateway-compatible pAAVS1-P-CAG-DEST vector from Knut Woltjen (RRID:Addgene_80490). The resulting vector (pAAVS1-CAG-DEST-DTA-TK) enabled constructs cloned into the attR sites to be driven by the strong, constitutive CAG (CAGGS) promoter [36].

Prior to targeting hiPSCs, all plasmid DNA was purified by Triton X-114 isothermal extraction as described previously [31]. Cell lines were then targeted at the *AAVS1* locus to generate AAVS1-TM-MCHR1(3xCTS)-mScarlet-APEX2 and AAVS1-TM-mScarlet-APEX2 cell lines using zinc finger nuclease (ZFN)-mediated genome editing, as described previously [37]. Briefly, KOLF2.1J and WTC-11 hIPSC were electroporated using the P3 Primary Cell 4D-Nucleofector kit at a 2:1 ratio of *AAVS1* targeting vector:ZNF DNA per reaction. A plasmid-encoding ZFN targeting the *AAVS1* locus (pCMV-ZFN-AAVS1) was a gift from Dmitriy Mazurov (RRID:Addgene_89707) [38]. KOLF2.1J was targeted with both constructs, whereas the WTC-11 line was targeted only with the AAVS1-TM-MCHR1(3xCTS)-mScarlet-APEX2 construct. After nucleofection, cells were plated onto plates coated with 25 µg/mL Synthemax in StemFlex media supplemented with 10 µg/mL Rock Inhibitor Y-27632 and 1 μM Alt-R™ HDR (homology-directed repair) Enhancer V2 and cultured in a 32 °C/5% CO_2_ incubator to cold shock cells and enhance HDR efficiency [39]. The media were then replaced with StemFlex media without Y-27632 or HDR enhancer the next day (24 h) and the cells were moved back to the 37 °C/5% CO_2_ incubator after 3 days (72 h). When cultures reached approximately 70% confluence, media were changed daily for five days with StemFlex supplemented with 0.5 μg/mL puromycin dihydrochloride to select for stably modified cell clones. After allowing resulting single-cell-derived clones to expand, clones were placed into 96 well plates, expanded, and tested for stable integration at the *AAVS1* locus by PCR amplification from genomic DNA as previously described [40].

### 2.7. Immunofluorescence and Imaging

To visualize the HaloTag in live cells, cultures were incubated with 200 nM Janelia Fluor HaloTag Ligand [41] JF503/635i or 1μM Promega HaloTag Alexa Fluor 488 Ligand for 15 min at 37 °C. Cells were washed 2 times with media and 1 time with PBS, then fixed as described below. For immunofluorescence, neurons derived from hiPSCs, and IMCD3, 3T3, and RPE1 cell lines were cultured on PhenoPlate 96-well microplates, fixed in 4% paraformaldehyde for 20 min at room temperature, and washed once with phosphate-buffered saline (PBS). IMCD3, 3T3, and RPE1 cell lines were then permeabilized with cold methanol at −20 °C for 10 min and rinsed three times with PBS. Cells were then incubated overnight at 4 °C with primary antibodies diluted in a blocking solution composed of PBS with 0.1% Triton X-100 (PBST) and 1% sterile-filtered normal donkey serum (NDS). Cells were then washed three times with PBS and incubated with secondary antibodies diluted in blocking solution for 2 h at room temperature. Finally, cells were washed three times with PBS and incubated for 5 min at room temperature with 300 μM of 4′,6-diamidino-2-phenylindole (DAPI) in PBS before being washed with PBS and immediately imaged, or stored in PBS with 0.1% sodium azide.

Images were acquired with the Perkin Elmer Opera Phenix Plus High-Content Screening System (Seer Green, UK) with a 40× water objective, with 10–15 optical planes per Z-stack at 1 μm intervals. Images were processed using the ImageJ software (Version 2) to create single maximum projection image. Some photomicrographs were background-subtracted to aid in visualization without adjusting gamma values. Some figure elements were prepared with the help of BioRender.com (accessed on 3 July 2024), and figures were assembled using Affinity Designer software (Version 2.3.1) (Table 1).

### 2.8. Quantification of Ciliary Labeling Efficiency

Ciliary targeting efficiency in IMCD3, 3T3, and RPE1 cells was assessed in all cells that had detectable fluorescence from the transfected construct, had a primary cilium immunopositive for ARL13B, and were located completely within imaging field borders. Among these cells, we defined ‘cilia targeting’ as the percentage of cells in which mScarlet fluorescence was detected in either just the ARL13B+ cilium or the ARL13B+ cilium and other cellular compartments. In hiPSC-derived neurons stably targeted with the cilia reporter construct (or transmembrane control constructs), we immunostained cultures for ARL13B (488 or 647 nm), counterstained with DAPI (405 nm), exposed cells to HaloTag ligand (488 or 635 nm), and imaged these channels and endogenous mScarlet (555 nm) signals. We then quantified the fraction of ARL13B+ cilia that co-expressed either the HaloTag ligand or mScarlet, and also quantified the fraction of HaloTag ligand positive or mScarlet positive ciliary structures that were immunopositive for ARL13B.

### 2.9. Cilia Length Quantification

Hypothalamic neurons were transferred to BrainPhys medium containing B27 and N2 Supplement and 1x penicillin-streptomycin 48 h prior to incubation with 25 mM LiCl and 50 mM LiCl for 1 h, 2 h, or 4 h, or 4 mg/mL colchicine for 1 h. Cilia length in hiPSC-derived neurons stained with DAPI (405 nm) and HaloTag ligand (488 nm) was measured via an automated analysis pipeline in Harmony high-content imaging and analysis software (Perkin Elmer, Seer Green, UK; version 4.9). Briefly, a fixed-intensity threshold was applied to every image in the 488 (HaloTag ligand) channel to exclude background noise, followed by the automated detection of objects with an area bigger than 4 µm^2^, length smaller than 20 µm, roundness value lower than 0.8, and intensity value higher than 36. Objects falling on imaging field borders were excluded from analysis. Finally, cilia number and the length of HaloTag ligand+ objects (in microns) were measured. Detailed analysis workflows are available upon request.

### 2.10. Statistical Analysis

GraphPad Prism (Version 10.2.0) was used for statistical analysis. Data are presented as mean ± standard error of the mean (SEM) or individual values ± SEM. ANOVA followed by Bonferroni post hoc test or Kruskal–Wallis test followed by Dunn’s post hoc test were used to compare more than two datasets. Mann–Whitney test was used to compare two datasets. *p* values < 0.05 were considered to be statistically significant.

## 3. Results

### 3.1. Identification of CTS Sufficient for Ciliary Targeting in Human Neurons

**Figure 1:**
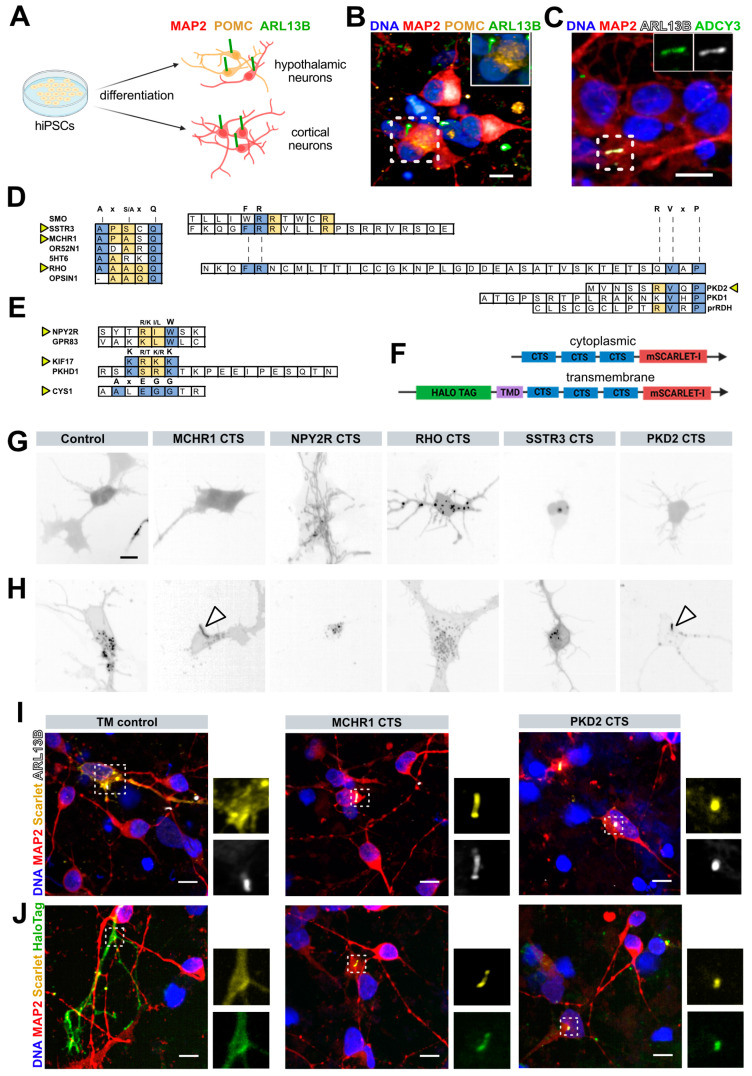
Targeting of transmembrane constructs to primary cilia in human neurons. (**A**) Schematic showing the differentiation of hiPSC into hypothalamic neurons (including POMC) and cortical neurons that have primary cilia expressing the cilia-enriched markers ARL13B (green and grey) and ADCY3 (green); (**B**) ARL13B+ (green) primary cilia project from the cell bodies of hypothalamic neurons, including those immunopositive for POMC (yellow); (**C**) Cortical neuron example cilia co-expressing ARL13B (grey) and ADCY3 (green) (also seen in hypothalamic neurons); (**D**) Sequences shown to be necessary for ciliary targeting from different proteins, where the consensus sequence is in bold, conserved residues are in blue, and less conserved residues are in yellow. Sequences selected in this study are indicated with arrowheads; (**E**) As in (d) but for distinct CTSs; (**F**) Schematic of construct design, where a 3x array of candidate CTSs followed by mScarlet with or without an N-terminal signal peptide, HaloTag, and transmembrane domain were used to test ciliary targeting; (**G**,**H**) Representative images of mScarlet signal from transiently transfected neurons showing the relative lack of ciliary targeting with cytoplasmic constructs (**G**), but ciliary localisation with CTSs derived from MCHR1 and PKD2 in transmembrane constructs ((**H**), arrowheads); (**I**) Sequences from MCHR1 and PKD2 drove localisation of mScarlet (yellow) to structures immunopositive for ARL13B (grey), and could also be visualized with fluorescent HaloTag ligands (green) (**J**). Insets show cilia at higher magnification. Scale bars represent 10 µm in (**B**,**C**,**G**,**I**,**J**).

We set out to find new methods to label primary cilia in live human cortical and hypothalamic neurons derived from hiPSCs (Figure 1A and Figure S1A,B) and ultimately use these tools to study the function of primary cilia. To confirm that these neurons are indeed ciliated, we first differentiated the ‘reference’ hiPSC line KOLF2.1J [24] into hypothalamic and cortical neurons as previously described [25,26,28] and immunostained these cultures for the neuronal marker microtubule-associated protein 2 (MAP2) and the primary cilia markers ADP ribosylation factor like GTPase 13B (ARL13B) and adenylate cyclase 3 (ADCY3). We found that hiPSC-derived cortical and hypothalamic neurons, including appetite-suppressing proopiomelanocortin (POMC) neurons, had cilia-shaped structures about 3–6 microns in length that projected from neuronal cell bodies, and were immunopositive for primary cilia markers (Figure 1B,C). Next, we set out to label these neuronal cilia with existing fusion constructs of fluorescent reporters and protein domains reported to mediate ciliary targeting. We therefore introduced the Cilia-APEX [22] expression plasmid, in which ciliary localisation is mediated by the N-terminal NPHP3 CTS, into hiPSC-derived neurons and the ciliated IMCD3 cell line by transient transfection. While we confirmed its ciliary localisation in IMCD3 cells, the construct failed to localize to primary cilia in hiPSC-derived human neurons across a broad range of tested plasmid concentrations, transfection methods, or neuronal maturation time points (Figure S1C,D).

We hypothesized that this lack of ciliary labeling in human neurons was due to different cell type-specific mechanisms regulating protein trafficking to the ciliary base, or mechanisms regulating ciliary entry. Certain amino acid motifs are recurrently described for ciliary targeting in certain cell types and protein contexts (Figure 1D,E), including Ax[S/A]xQ [17], VxP [42,43,44,45], FR [43,46], [R/K][I/L]W [23], KRKK [23,47], KTRK [48], and AxEGG [49]. We selected CTSs from seven genes to test the ability of each of these motif types to mediate ciliary targeting in human neurons (Table 2).

Specifically, we selected regions containing the Ax[S/A]xQ motif from MCHR1 and SSTR3 [17,50] and regions containing the [R/K][I/L]W motif from NPY2R [23], since all three of these GPCRs localize to primary cilia in hypothalamic neurons [51]. We also tested candidate CTSs containing a VxP motif from PKD2 [44], and a CTS from RHO that contains three motifs (Ax[S/A]xQ, FR, and VxP) [42]. Finally, the CTS motifs KRKK and AxEGG were selected from KIF17 and CYS1 [47,49]. We did not consider candidate CTSs from 5HT6 and PKHD1 due to their lower sequence homology between human and mice [17,48]. We selected 3–5 amino acids on either side of the described core CTS motif, and reasoned that a 3x tandem array of these candidate sequences separated by a short flexible linker (GGGGS) would increase the likelihood of engagement with ciliary localisation machinery [52]. We omitted the linkers for the CTS from PKD2 due to its relatively large size.

We then cloned these CTS arrays upstream of the bright monomeric red fluorescent protein mScarlet-I to enable visualization of the construct in live cells [53,54]. Since ciliary targeting mechanisms are likely distinct for cytosolic and transmembrane or lipid-associated proteins, and it is unclear how isolated CTSs taken from different proteins would act in the context of a fusion reporter construct, we generated both cytoplasmic and transmembrane versions of most of these constructs [9] except CYS1 and KIF17. To generate the transmembrane versions, we included an N-terminal IL2 signal peptide and HaloTag to facilitate extracellular fluorescent labeling [55], a GGGS linker and synthetic transmembrane domain consisting of 22 leucine residues and four basic residues to stabilize the protein in the membrane and promote export through the biosynthetic secretory pathway [34,56,57], and GS residues just upstream of the CTS array to localize putative ciliary targeting sequences close to the plasma membrane (Figure 1F, Table 2). We then electroporated these cytoplasmic and transmembrane constructs into hiPSC-derived hypothalamic neurons and screened for the localisation of mScarlet cilia-like structures. We found that none of the cytoplasmic constructs we tested showed apparent ciliary enrichment (Figure 1G and Figure S1E) but that two of the transmembrane constructs containing CTSs derived from MCHR1 or PKD2 localized preferentially to cilia-like structures in transfected cells (Figure 1H).

To confirm these findings, we fixed and immunostained cultures for neuronal and ciliary markers. We found that while mScarlet was localized to the plasma membrane for transmembrane control constructs lacking CTS arrays (TM control), mScarlet co-localized with ARL13B in cells transfected with constructs containing MCHR1- or PKD2-derived CTSs. Since these constructs also contain an extracellular HaloTag, we added fluorescent HaloTag ligands and found that this fluorescent signal also co-localised with both mScarlet and ciliary markers (Figure 1I,J). Together, these findings indicate that primary cilia in live human neurons can be visualized with both an intracellular fluorescent protein and a variety of extracellular HaloTag ligands.

### 3.2. Ciliary Targeting across Diverse Ciliated Cell Lines

**Figure 2:**
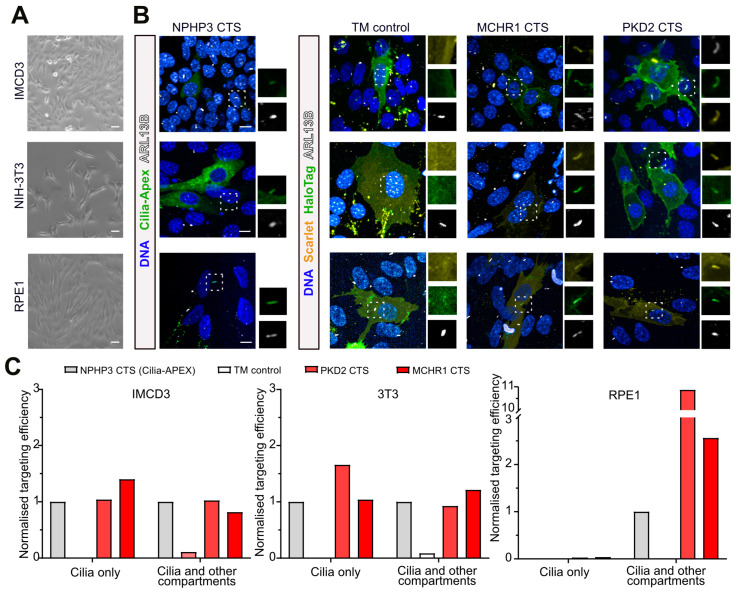
CTSs from MCR1 and PDK2 are sufficient for ciliary targeting in diverse cell lines in the context of a TMD. (**A**) Phase contrast photomicrographs of ciliated IMCD3, NIH-3T3, or RPE cell lines used to test for ciliary localisation by transient transfection. (**B**) Representative images of the subcellular localisation of experimental constructs as revealed by endogenous GFP fluorescence in cells transfected with NPHP3-containing Cilia-APEX construct, or mScarlet signal in cells transfected with control transmembrane (TM), or MCHR1- or PKD2-derived CTS-containing constructs. Scale bar is 100 µm for all images in (**A**) and 10 µm in (**B**). (**C**) Quantification of ciliary localisation for each tested cell line and constructs, normalized to the percentage of Cilia-APEX transfected cells where the fluorescent protein was exclusively observed in cilia, or observed in cilia and other cellular compartments, with the exception of the ‘cilia only’ category for RPE1 cells where no cells were identified for Cilia-APEX, n = 139 ± 49 (IMCD3), 50 ± 16 (3T3), and 81 ± 41 (RPE1) averaged quantified cells per condition.

Since the CTS arrays we tested from MCHR1 and PKD2 were sufficient to drive ciliary localisation in hiPSC-derived neurons, we next wondered whether constructs containing these sequences would localize to cilia in other cell lines, and how their performance would compare to the ‘Cilia-APEX’ construct encoding a fusion of the first 203 residues of NPHP3 [21], GFP, and the ascorbate peroxidase APEX to facilitate ciliary proximity proteomics [22,58,59] (Figure S2A). To replicate published studies, we first confirmed the ciliary targeting of this construct following transient transfection in serum-starved inner medullary collecting duct epithelial (IMCD3), mouse embryonic fibroblasts (NIH-3T3), and retinal pigment epithelial (RPE1-hTERT) cell lines (Figure 2A). Specifically, we fixed cell lines two days after transfection and immunostained for ARL13B to identify which cells were ciliated. We then identified ciliated cells with visible plasmid-derived fluorescence and split them into three categories depending on whether fluorescence was exclusively localized to ARL13B-expressing primary cilia, localized to both primary cilia and other cellular compartments, or present in other cellular compartments but not detectable in primary cilia (Figure S2B).

In agreement with previous reports, we found that Cilia-APEX containing the NPHP3 CTS localized to primary cilia in all tested cell lines, albeit at lower efficiency in RPE1 cells and less than 100% efficiency in all cell lines, likely due the variability associated with transient transfection (Figure 2B). In parallel, we transfected the same cell lines with plasmids encoding our transmembrane constructs that either lacked a CTS (TM control), or contained MCHR1-3xCTS or PKD2-3xCTS. We then quantified the fraction of cells with exclusive or partial ciliary localization, as we had for Cilia-APEX, and found that MCHR1-3xCTS and PKD2-3xCTS performed at least as well (Figure 2C and Figure S2B), suggesting they are sufficient for ciliary targeting in a range of ciliated cell types, including human neurons.

### 3.3. Generation of Stably Cilia-Tagged Human Pluripotent Stem Cell Lines

**Figure 3:**
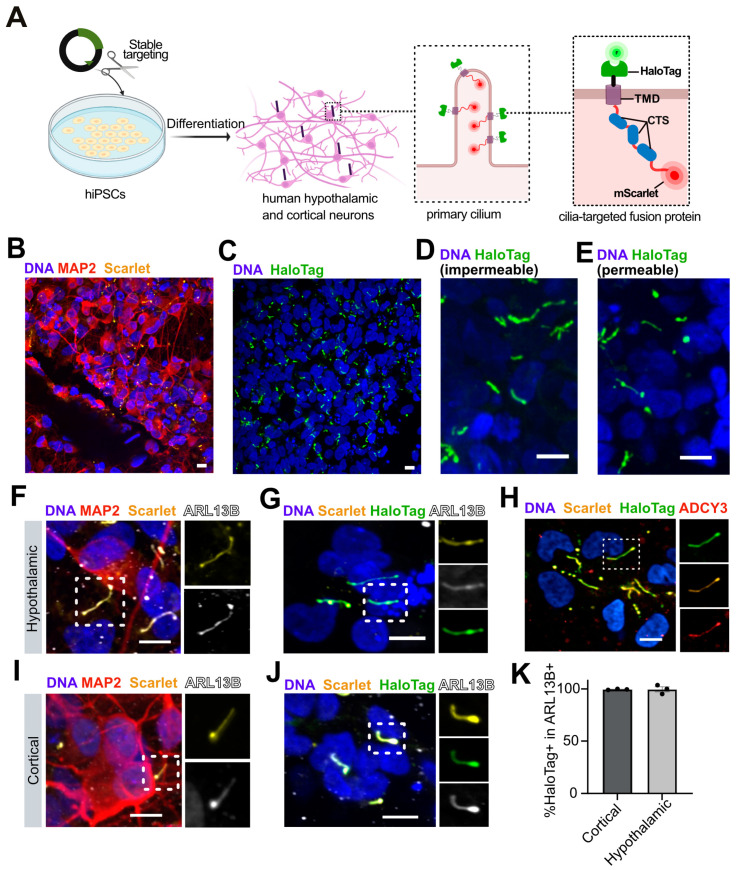
A stable hiPSC cell line constitutively expressing a primary cilia reporter. (**A**) Experimental schematic for stable targeting of human *AAVS1* locus to introduce a constitutively expressed ciliary reporter. (**B**,**C**) Low-magnification images of primary cilia in hiPSC-derived hypothalamic neurons visualized by mScarlet (**B**) or a fluorescent HaloTag ligand (**C**). (**D**,**E**) Higher magnification images of cultures in (**C**) where cilia are visualized with cell-impermeable (**D**) or permeable (**E**) HaloTag ligands to confirm the specificity of the reporter signal. (**F**–**H**) In hiPSC-derived MAP2+ (red) hypothalamic neurons, ARL13B+ primary cilia (grey) can be visualized by both endogenous fluorescence of mScarlet (yellow) (**F**), and fluorescent HaloTag ligand (green) (**G**) in cilia that are immunopositive for ADCY3 (red) (**H**). (**I**,**J**) Similar results are seen in hiPSC-derived cortical neurons. Scale bars represent 10 µm in panels (**B**–**J**). (**K**) Fraction of ARL13B+ cilia that also are labeled by the HaloTag ligand in cortical and hypothalamic neurons. N = 3 technical replicates (each dot represents one technical replicate), n = 25 ± 3 (hypothalamic), and 268 ± 56 (cortical) ARL13B+ structures per replicate.

We hypothesized that the variable ciliary targeting efficiency we observed upon transient transfection could be improved by stably introducing a single copy of our ciliary targeting construct into a genetic ‘safe harbor’ locus. By targeting hiPSC lines, primary cilia could be labeled in any of their differentiated progeny, including cortical and hypothalamic neurons. We therefore extended the MCHR1-3xCTS transmembrane construct with a C-terminal ascorbate peroxidase (APEX2) to facilitate proximity labeling in future studies [22,59], and cloned it downstream of a CAG promoter into a targeting vector for the *AAVS1* safe harbor locus that facilitates transgene expression in hPSCs and their differentiated progeny [60] (Figure 3A and Figure S3A). We then introduced this construct, or a TM control construct lacking the MCHR1-3xCTS, into the deeply characterized KOLF2.1J hiPSC line that differentiates well into neural cell types [24]. We also targeted the *AAVS1* locus in a clone of the WTC-11 hiPSC line [25] that had been previously modified to express neurogenin2 (NGN2) in a doxycycline-inducible manner to efficiently drive cortical-like neuronal differentiation [28]. Upon drug selection, we picked clones, confirmed *AAVS1* targeting by PCR, and further modified targeted lines on the KOLF2.1J background by introducing a doxycycline-inducible NGN2 expression construct by PiggyBac-mediated stable integration.

### 3.4. Efficient Ciliary Targeting in Stable Cell Lines

To assess the subcellular localisation of ciliary reporter constructs, we differentiated clones of KOLF2.1J and WTC-11 carrying both the stably integrated NGN2 transgene and either the TM or MCHR1-3xCTS into hypothalamic neurons (Figure 3B). Upon imaging live cells, we found that the mScarlet signal did not localize to cilia in cells expressing the TM construct (Figure S3B,C) but showed clear cilia-like localisation in cells targeted with constructs containing MCHR1-3xCTS (Figure 3B,C). Upon the addition of fluorescent HaloTag ligands to live cells, we observed a bright and exclusively cilia-like fluorescence pattern for cells expressing the MCHR1-3xCTS (Figure 3C), regardless of whether we added cell-permeable or cell-impermeable ligands (Figure 3D,E).

The expression pattern we observed appeared ciliary, but this might only be labeling a subset of all cilia or might be specific to hypothalamic differentiation. We therefore differentiated cell lines to both cortical and hypothalamic neurons and fixed and immunostained cultures for the cilia-enriched markers ARL13B and/or ADCY3 (Figure 3F–J). After 10 days of differentiation, nearly all cells (93.4 ± 4.6%) from cortical differentiation had adopted neuronal morphology and were immunopositive for the neuronal marker gene MAP2 (Figure 3I).We then quantified the fraction of ARL13B immunopositive structures that were also positive for mScarlet and the Halo ligand (Figure 3K), as well as the converse (mScarlet and HaloTag ligand-expressing structures that were also immunopositive for ARL13B, Figure S3D). We observed nearly complete co-localisation (>99%) of our construct with ARL13B+ primary cilia (Figure 3K). We observed very similar results when we repeated this analysis in hiPSC differentiated to hypothalamic neurons (Figure 3I–K), including ciliated POMC neurons (Figure S3E).

Finally, since the overexpression of ciliary proteins has been shown to increase cilia length [61], we quantified the length of primary cilia in hypothalamic neurons derived from clones of hiPSCs derived from the same parental cell line and modified to carry either the MCHR1-3XCTS or the TM construct. Specifically, we differentiated cell lines in parallel, immunostained them for ARL13B, and manually quantified the length of ARL13B+ primary cilia. We found that cilia from cells expressing MCHR1-3xCTS tended to be longer than those in the TM control line (Figure S3F,G), suggesting that care should be taken when interpreting the absolute lengths of primary cilia carrying this construct. Overall, the cell lines we generated permit cilia to be studied and manipulated in a wide range of human cell types that can be generated from hiPSCs.

### 3.5. Image-Based Screening for Regulators of Primary Cilia Length (PCL)

**Figure 4:**
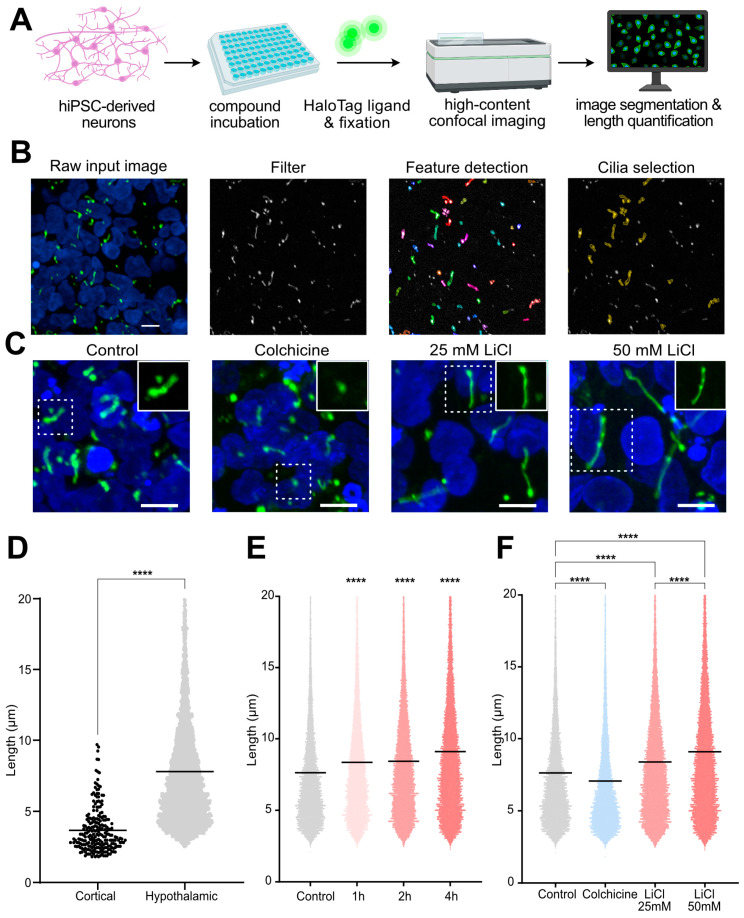
Modulation of primary cilia length (PCL) in human neurons. (**A**) Experimental schematic for the automatic confocal image-based quantification of drug-induced changes in PCL in human neuronal cilia visualized by HaloTag ligand. (**B**) Representative images of cell nuclei (blue) and fluorescent Halo ligand (green) that are filtered by thresholding prior to automatic segmentation and selection of objects that meet criteria expected of primary cilia, such as minimal area and roundness. Scale bars represent 10 µm. (**C**) Representative images from neuronal cultures treated with vehicle, 4 mg/mL colchicine, or 25 or 50 mM LiCl, where nuclei are in blue and the Halo ligand is in green. Scale bars represent 10 µm.(**D**) PCL measurements in hiPSC-derived cortical and hypothalamic neurons, where each data point represents one primary cilium. Bar indicates mean cilia length. n = 235 and n = 5038 quantified structures/replicate for cortical and hypothalamic, respectively. **** *p* < 0.0001 by Mann–Whitney test. (**E**) Time-dependent increase in measured PCL in response to 50 mM LiCl. (**F**) PCL changes in response to treatment with vehicle, 4 mg/mL colchicine for 1 h, or 25 or 50 mM LiCl for 4 h. N = 3 technical replicates, 30 fields/replicate, and 7850 ± 434 quantified structures per replicate. Bar indicates mean cilia length. **** *p* < 0.0001 by Kruskal–Wallis test.

Since primary cilia can be readily visualized in live human neurons in our stably targeted cell lines, we reasoned they would be ideal to characterize human diseases that affect primary cilia structure, and identify factors that regulate primary cilia length (PCL). Ciliary diseases can alter PCL, and in healthy cells, PCL dynamically changes in response to a variety of exogenous signals that impact actin and microtubule polymerization and other pathways, although the precise mechanisms and functional consequences of PCL changes remain poorly understood [62]. To test if PCL can be measured in hiPSC-derived cortical and hypothalamic neurons, we imaged cultures labeled with fluorescent HaloTag ligand on the Opera Phenix automated confocal microscope. The bright and specific signal of this ligand in monolayers of adherent neurons allowed primary cilia to be readily detected and measured in an automated analysis pipeline (Figure 4A). Specifically, we captured confocal stacks, took maximum intensity projections, and then automatically detected primary cilia and quantified their length (Figure 4B). This automated pipeline enabled thousands of cilia (7850 ± 434) to be measured in each well of a 96-well plate.

To test whether this analysis pipeline can be used to measure changes in PCL, we treated hypothalamic neuron cultures with lithium chloride (LiCl), which is known to increase PCL in many cell types including neurons [63], or with colchicine, an inhibitor of microtubule polymerisation that may destabilize cilia [64,65] (Figure 4C). Since cultures of hypothalamic neurons had a higher fraction of ciliated cells and longer average cilia (7.64 ± 0.14 µm) than cortical neurons in the conditions we tested (Figure 4D), we treated only hypothalamic cultures with vehicle or 25 or 50 mM LiCl for 1, 2, or 4 h, before adding fluorescent HaloTag ligands and fixing cells with 4% PFA (Figure 4C and Figure S4). We found that both 25 mM and 50 mM LiCl produced significant and time-dependent increases in PCL over the 4 h treatment time course (Figure 4E and Figure S4B). Next, we selected the 4 h time point and quantified changes in the lengths of a total 90,861 cilia in cultures treated with LiCl and 4 mg/mL colchicine for 1 h. We found that primary cilia in wells treated with colchicine were significantly shorter than vehicle-treated controls (*p* < 0.0001), whereas primary cilia in wells treated with 25 or 50 mM LiCl were significantly longer (*p* < 0.0001) (Figure 4F). We did not observe apparent changes in primary cilia number per well (Figure S4A). Together, these results demonstrate the utility of the cilia reporter cell line for measuring PCL and pave the way for larger-scale studies to screen for factors that regulate PCL across diverse cell types.

## 4. Discussion

We found that while commonly used CTS failed in human neurons, we could identify two small CTSs that were sufficient to drive ciliary localisation of a fluorescent construct in every ciliated cell line we tested using transient transfection, and worked at efficiencies approaching 100% in stably targeted cell lines. Here, we discuss the potential uses of the stable hiPSC cell line to study the biology of primary cilia in health and disease, the reasons these sequences were effective in neurons while others were not, and the strengths and limitations of our study.

The construct we stably targeted to hiPSCs permits live imaging of cilia via mScarlet fluorescence or fluorescent Halo ligands, pull-down or other manipulation of cilia via the extracellular HaloTag, and proximity proteomics of primary cilia. We also demonstrate that one can test for factors that alter primary cilia length by confirming the known lengthening effects of LiCl, and showing that colchicine treatment is sufficient to reduce primary cilia length under these conditions. The bright and specific ciliary staining obtained by the HaloTag ligand improves upon cilia identification using immunostaining in both its high signal to noise ratio and its ability to be applied to live cells rather than fixed cultures. These and other analyses made possible by stable ciliary reporter cell lines could be extended to any cell type that can be generated from hiPSCs to characterize alterations in primary cilia associated with signaling or disease state.

In our hands, a Cilia-Apex construct containing the myristoylation site and coiled-coil domains of NPHP3, previously reported to mediate ciliary targeting, ref. [21] did not show this activity in hiPSC-derived human neurons. We speculate that there are cell type-specific mechanisms that are either less efficient or absent in the neurons we tested. The similar features of the two successful constructs containing CTSs derived from MCHR1 and PKD2 sheds some light on these targeting mechanisms, as both contain a CTSs also derived from transmembrane proteins, suggesting both are required for membrane protein sorting to the primary cilium. Previous studies suggest that BBS proteins may be important for MCHR1 ciliary targeting, since this receptor accumulates in cytoplasmic puncta in *BBS2* and *BBS4* double knockout mutant mice [66]. Future immunoprecipitation experiments with the CTSs from this study could reveal shared interaction partners.

It is not clear why other tested CTSs did not work in our system. The sequence from KIF17 may have been insufficient when tested without the kinesin motor that appears to promote its association with importin-β2 and transport across the ciliary transition zone [47]. The CTS in NPHP3 and CYS1 both require myristoylation [21,49], and although these sequences were included in the sites we tested, it is possible that this post-translational modification did not proceed efficiently in iPSC-derived neurons. RHO requires a complex of TNPO1 and RAB8 [67] and/or dual Ax(S/A)xQ motifs for ciliary targeting, and the human RHO CTS tested contains only one of these motifs [68]. NPY2R and SSTR3 both localize to neuronal primary cilia, but the conformation of the isolated CTS might be different than in the native protein, additional sequence context might be necessary for the isolated CTSs to engage with the ciliary targeting machinery, or perhaps post-translational modifications in the CTS differ between the native protein and our artificial construct.

## 5. Limitations of Study

We could not exhaustively test all potential CTSs and cannot exclude the possibility that a more potent or universally effective CTS remains to be discovered. To increase our chances of success, we tested CTS in a 3x array of putative CTS with a ‘core’ sequence important for ciliary targeting and flanked with surrounding residues to provide sequence context. Future studies could test whether a single copy of the CTS is sufficient to mediate ciliary targeting, or systematically mutate or delete residues in the tested CTS to identify those that are necessary for the observed targeting. These studies might enable more compact or effective CTS to be designed and provide insights into the mechanisms of ciliary targeting.

Of the constructs we tested that included a TMD, the candidate CTSs were separated from the membrane by a short sequence (KRKRGS). As the TMD was required for ciliary targeting, it is possible that the artificial nature of our TMD (string of leucine residues), the positively charged residues following this sequence, or the proximity of the CTS to the membrane contributed to the observed activity. Similarly, we did not test constructs targeted to the membrane by lipid modification, which could engage distinct mechanisms of ciliary localisation. The IL2 signal peptide is widely used to target proteins to the secretory or transmembrane pathways [69], and our staining with cell-permeable and cell-impermeable Halo ligands suggests that our construct efficiently inserted into the plasma membrane the HaloTag facing the outside of the cell. Systematically testing other signal peptides might shed light onto their cooperative or antagonistic interactions with cytoplasmic CTSs.

Although we tested two different methods of generating neurons from hiPSCs and obtained similar results on two different genetic backgrounds, it is possible that some of the effects we observed could be due to the relatively immature state of cells derived from hiPSCs. Finally, when generating stable cell lines, we targeted a single copy of the construct to the *AAVS1* locus under the control of the strong and constitutive CAG promoter, and acknowledge that its presence at the cilium might alter endogenous protein localisation, either by physically crowding out ciliary proteins or competing for access to the ciliary localisation and transport machinery. Since the overexpression of the construct via a strong constitutive promoter (CAG) appears to increase primary cilia length, users may wish to use weaker promoters or other approaches to mitigate this effect.

## 6. Conclusions

Primary cilia play key roles in cellular signaling, but there have been relatively few tools developed to study them in live cells. To address this challenge and enable the study of primary cilia in live human neurons, we screened candidate ciliary targeting sequences and identified a short sequence of amino acids from MCHR1 that is sufficient to target transmembrane constructs to primary cilia across multiple ciliated cell lines. The versatility of the short CTS we describe should enable groups to target constructs of their interest to primary cilia in their preferred cell types and model systems. The short size of the CTS we describe makes it an attractive candidate for generating fusion proteins that must remain compact due to steric limitations or viral genomes for use in animal models. We then generated a hiPSC line stably expressing this construct that strongly and selectively labels primary cilia, enabling functional studies of primary cilia in healthy and diseased states across a wide variety of live human cell types. We demonstrate the value of this tool by characterizing changes in primary cilia lengths in response to experimental compounds. We hope that the resources reported in this study will aid other groups researching primary cilia.

## Figures and Tables

**Table 1:** Antibodies used in this study.

Primary Antibodies		
Antibody	Dilution	Catalogue
anti-MAP2	1:2000	Abcam (Cambridge, UK) ab5392
anti-ARL13b	1:700	Proteintech (Manchester, UK) 17711-1-AP
anti-ADCY3	1:500	SantaCruz (Wembley, UK) sc-518057
anti-RFP	1:500	Synaptic System (Goettingen, Germany) 409 011
Anti-POMC (ACTH/αMSH)	1:5000	Prof. Anne White (University of Manchester, UK)
**Secondary Antibodies**		
**Antibody**	**Dilution**	**Catalogue**
Alexa donkey anti-rabbit 488	1:500	Thermo Fisher Scientific (Altrincham, UK) A21206
Alexa donkey anti-mouse 555	1:500	Thermo Fisher Scientific (Altrincham, UK) A31570
Alexa donkey anti-chicken 647	1:500	Stratech (Ely, UK) 703-606-155

**Table 2:** Candidate ciliary targeting sequences (CTS) from candidate genes include core motif sequences (bold) and flanking residues. Selected sequences (right-hand column) were cloned into a 3x array for testing in hiPSC-derived neurons and other ciliated cell types.

Gene Name	Protein Reference Sequence	Candidate CTS from Literature	References	Selected CTS Position	Selected CTS Sequence
*CYS1* (Cystin 1)	NP_001032237.1	MGSGSSRSSR; RRRRS; AALEGGTR	[49]	1–44, disordered region. The second residue (G) is a myristoylation site	**MGSGSSRSSR**TL**RRRRS**PESLPAGPGA**AALEGGTR**RRVPVAAAE
*KIF17* (Kinesin family member 17)	NP_001116291.1	KRKK	[47]	1011–1022, disordered region	FTKA**KRKK**SKSN
*MCHR1* (Melanin concentrating hormone receptor 1)	NP_005288.4	APASQ	[17]	235–248, third cytoplasmic loop	TSSV**APASQ**RSIR
*NPY2R* (Neuropeptide Y receptor Y2)	NP_000901.1	[R,K][I,L]W	[23]	237–248, third cytoplasmic loop	SYT**RIW**SKLKN
*PKD2* (Polycystin 2/PC2)	NP_000288.1	SxRVxP included in aa 5–72	[43,44]	1–76, disordered cytoplasmic domain	MVN**SSRVQPQ**QPGDAKRPPAPRAPDPGRLMAGCAAVGASLAAPGGLCEQRGLEIEMQRIRQAAARDPPAGAAASPS
*RHO* (Rhodopsin)	NP_000530.1	AAAQQ; FR; SQVAPA	[17,42]	228–242, third cytoplasmic loop (partial), and 310–348, last cytoplasmic loop	FTVKE**AAAQQ**QESATNKQ**FR**NCMLTTICCGKNPLGDDEASATVSKTETS**QVAPA**
*SSTR3* (Somatostatin receptor 3)	NP_001042.1	APSCQ	[17,50]	232–257, third cytoplasmic loop	KVRSAGRRVW**APSCQ**RRRRSERRVTR

## Data Availability

The raw data supporting the conclusions of this article and relevant resources will be made available by the authors on request. For the purpose of open access, the authors have applied a CC-BY public copyright license to any Author Accepted Manuscript version arising from this submission.

## Supplementary Materials

The following supporting information can be downloaded at: https://www.mdpi.com/article/10.3390/cells13131156/s1, Figure S1: Neuronal differentiation of hiPSCs and testing of ciliary localisation.; Figure S2: Cilia targeting constructs design and their localisation in diverse ciliated cell lines; Figure S3: Design and testing of ciliary reporter cell lines; Figure S4: Effects of LiCl and colchicine on primary cilia length in human neurons; Key Resources Table.

## References

[1] Anvarian Z., Mykytyn K., Mukhopadhyay S., Pedersen L.B., Christensen S.T. (2019). Cellular signalling by primary cilia in development, organ function and disease. Nat. Rev. Nephrol..

[2] Wang Y., Bernard A., Comblain F., Yue X., Paillart C., Zhang S., Reiter J.F., Vaisse C. (2021). Melanocortin 4 receptor signals at the neuronal primary cilium to control food intake and body weight. J. Clin. Investig..

[3] Davenport J.R., Watts A.J., Roper V.C., Croyle M.J., van Groen T., Wyss J.M., Nagy T.R., Kesterson R.A., Yoder B.K. (2007). Disruption of intraflagellar transport in adult mice leads to obesity and slow-onset cystic kidney disease. Curr. Biol..

[4] Brewer K.M., Brewer K.K., Richardson N.C., Berbari N.F. (2022). Neuronal cilia in energy homeostasis. Front. Cell Dev. Biol..

[5] Nachury M.V., Mick D.U. (2019). Establishing and regulating the composition of cilia for signal transduction. Nat. Rev. Mol. Cell Biol..

[6] Nakayama K., Katoh Y. (2020). Architecture of the IFT ciliary trafficking machinery and interplay between its components. Crit. Rev. Biochem. Mol. Biol..

[7] Van den Hoek H., Klena N., Jordan M.A., Alvarez Viar G., Righetto R.D., Schaffer M., Erdmann P.S., Wan W., Geimer S., Plitzko J.M. (2022). In situ architecture of the ciliary base reveals the stepwise assembly of intraflagellar transport trains. Science.

[8] Long H., Huang K. (2019). Transport of Ciliary Membrane Proteins. Front. Cell Dev. Biol..

[9] Nachury M.V., Seeley E.S., Jin H. (2010). Trafficking to the ciliary membrane: How to get across the periciliary diffusion barrier?. Annu. Rev. Cell Dev. Biol..

[10] Garcia-Gonzalo F.R., Reiter J.F. (2012). Scoring a backstage pass: Mechanisms of ciliogenesis and ciliary access. J. Cell Biol..

[11] Witzgall R. (2018). Golgi bypass of ciliary proteins. Semin. Cell Dev. Biol..

[12] Gonçalves J., Pelletier L. (2017). The Ciliary Transition Zone: Finding the Pieces and Assembling the Gate. Mol. Cells.

[13] Hu Q., Milenkovic L., Jin H., Scott M.P., Nachury M.V., Spiliotis E.T., Nelson W.J. (2010). A septin diffusion barrier at the base of the primary cilium maintains ciliary membrane protein distribution. Science.

[14] Jensen V.L., Leroux M.R. (2017). Gates for soluble and membrane proteins, and two trafficking systems (IFT and LIFT), establish a dynamic ciliary signaling compartment. Curr. Opin. Cell Biol..

[15] Kee H.L., Dishinger J.F., Blasius T.L., Liu C.-J., Margolis B., Verhey K.J. (2012). A size-exclusion permeability barrier and nucleoporins characterize a ciliary pore complex that regulates transport into cilia. Nat. Cell Biol..

[16] O’Connor A.K., Malarkey E.B., Berbari N.F., Croyle M.J., Haycraft C.J., Bell P.D., Hohenstein P., A Kesterson R., Yoder B.K. (2013). An inducible CiliaGFP mouse model for in vivo visualization and analysis of cilia in live tissue. Cilia.

[17] Berbari N.F., Johnson A.D., Lewis J.S., Askwith C.C., Mykytyn K. (2008). Identification of ciliary localization sequences within the third intracellular loop of G protein-coupled receptors. Mol. Biol. Cell.

[18] Guo J., Otis J.M., Suciu S.K., Catalano C., Xing L., Constable S., Wachten D., Gupton S., Lee J., Lee A. (2019). Primary Cilia Signaling Promotes Axonal Tract Development and Is Disrupted in Joubert Syndrome-Related Disorders Models. Dev. Cell.

[19] Jiang J.Y., Falcone J.L., Curci S., Hofer A.M. (2019). Direct visualization of cAMP signaling in primary cilia reveals up-regulation of ciliary GPCR activity following Hedgehog activation. Proc. Natl. Acad. Sci. USA.

[20] Moore B.S., Stepanchick A.N., Tewson P.H., Hartle C.M., Zhang J., Quinn A.M., Hughes T.E., Mirshahi T. (2016). Cilia have high cAMP levels that are inhibited by Sonic Hedgehog-regulated calcium dynamics. Proc. Natl. Acad. Sci. USA.

[21] Nakata K., Shiba D., Kobayashi D., Yokoyama T. (2012). Targeting of Nphp3 to the primary cilia is controlled by an N-terminal myristoylation site and coiled-coil domains. Cytoskeleton.

[22] Mick D.U., Rodrigues R.B., Leib R.D., Adams C.M., Chien A.S., Gygi S.P., Nachury M.V. (2015). Proteomics of Primary Cilia by Proximity Labeling. Dev. Cell.

[23] Loktev A.V., Jackson P.K. (2013). Neuropeptide Y family receptors traffic via the Bardet-Biedl syndrome pathway to signal in neuronal primary cilia. Cell Rep..

[24] Pantazis C.B., Yang A., Lara E., McDonough J.A., Blauwendraat C., Peng L., Oguro H., Kanaujiya J., Zou J., Sebesta D. (2022). A reference human induced pluripotent stem cell line for large-scale collaborative studies. Cell Stem Cell.

[25] Kreitzer F.R., Salomonis N., Sheehan A., Huang M., Park J.S., Spindler M.J., Lizarraga P., A Weiss W., So P.-L., Conklin B.R. (2013). A robust method to derive functional neural crest cells from human pluripotent stem cells. Am. J. Stem Cells..

[26] Chen H.-J.C., Mazzaferro S., Tian T., Mali I., Merkle F.T. (2023). Differentiation, Transcriptomic Profiling, and Calcium Imaging of Human Hypothalamic Neurons. Curr. Protoc..

[27] Mukhopadhyay S., Wen X., Chih B., Nelson C.D., Lane W.S., Scales S.J., Jackson P.K. (2010). TULP3 bridges the IFT-A complex and membrane phosphoinositides to promote trafficking of G protein-coupled receptors into primary cilia. Genes Dev..

[28] Fernandopulle M.S., Prestil R., Grunseich C., Wang C., Gan L., Ward M.E. (2018). Transcription Factor-Mediated Differentiation of Human iPSCs into Neurons. Curr. Protoc. Cell Biol..

[29] Tian R., Gachechiladze M.A., Ludwig C.H., Laurie M.T., Hong J.Y., Nathaniel D., Prabhu A.V., Fernandopulle M.S., Patel R., Abshari M. (2019). CRISPR Interference-Based Platform for Multimodal Genetic Screens in Human iPSC-Derived Neurons. Neuron.

[30] Flores E.L., Qi A., Reilly L., Santiana M., Ward M., Cookson M. (2022). piNDI Transcription Factor-NGN2 Differentiation of Human iPSC into Cortical Neurons Version 1 v1.

[31] Ma R., Zhao J., Du H.-C., Tian S., Li L.-W. (2012). Removing endotoxin from plasmid samples by Triton X-114 isothermal extraction. Anal. Biochem..

[32] Merkle F.T., Maroof A., Wataya T., Sasai Y., Studer L., Eggan K., Schier A.F. (2015). Generation of neuropeptidergic hypothalamic neurons from human pluripotent stem cells. Development.

[33] Kirwan P., Jura M., Merkle F.T. (2017). Generation and Characterization of Functional Human Hypothalamic Neurons. Curr. Protoc. Neurosci..

[34] Chen H., Kendall D.A. (1995). Artificial transmembrane segments. Requirements for stop transfer and polypeptide orientation. J. Biol. Chem..

[35] Nam H.-S., Benezra R. (2009). High levels of Id1 expression define B1 type adult neural stem cells. Cell Stem Cell.

[36] Oceguera-Yanez F., Kim S.-I., Matsumoto T., Tan G.W., Xiang L., Hatani T., Kondo T., Ikeya M., Yoshida Y., Inoue H. (2016). Engineering the AAVS1 locus for consistent and scalable transgene expression in human iPSCs and their differentiated derivatives. Methods.

[37] Urnov F.D., Rebar E.J., Holmes M.C., Zhang H.S., Gregory P.D. (2010). Genome editing with engineered zinc finger nucleases. Nat. Rev. Genet..

[38] Zotova A., Lopatukhina E., Filatov A., Khaitov M., Mazurov D. (2017). Gene Editing in Human Lymphoid Cells: Role for Donor DNA, Type of Genomic Nuclease and Cell Selection Method. Viruses.

[39] Skarnes W.C., Pellegrino E., McDonough J.A. (2019). Improving homology-directed repair efficiency in human stem cells. Methods.

[40] Santos D.P., Kiskinis E., Eggan K., Merkle F.T. (2016). Comprehensive Protocols for CRISPR/Cas9-based Gene Editing in Human Pluripotent Stem Cells. Curr. Protoc. Stem Cell Biol..

[41] Grimm J.B., English B.P., Chen J., Slaughter J.P., Zhang Z., Revyakin A., Patel R., Macklin J.J., Normanno D., Singer R.H. (2015). A general method to improve fluorophores for live-cell and single-molecule microscopy. Nat. Methods.

[42] Wang J., Deretic D. (2014). Molecular complexes that direct rhodopsin transport to primary cilia. Prog. Retin. Eye Res..

[43] Ward H.H., Brown-Glaberman U., Wang J., Morita Y., Alper S.L., Bedrick E.J., Gattone V.H., Deretic D., Wandinger-Ness A. (2011). A conserved signal and GTPase complex are required for the ciliary transport of polycystin-1. Mol. Biol. Cell.

[44] Geng L., Okuhara D., Yu Z., Tian X., Cai Y., Shibazaki S., Somlo S. (2006). Polycystin-2 traffics to cilia independently of polycystin-1 by using an N-terminal RVxP motif. J. Cell Sci..

[45] Luo W., Marsh-Armstrong N., Rattner A., Nathans J. (2004). An outer segment localization signal at the C terminus of the photoreceptor-specific retinol dehydrogenase. J. Neurosci..

[46] Chadha A., Paniagua A.E., Williams D.S. (2021). Comparison of Ciliary Targeting of Two Rhodopsin-Like GPCRs: Role of C-Terminal Localization Sequences in Relation to Cilium Type. J. Neurosci..

[47] Dishinger J.F., Kee H.L., Jenkins P.M., Fan S., Hurd T.W., Hammond J.W., Truong Y.N., Margolis B., Martens J.R., Verhey K.J. (2010). Ciliary entry of the kinesin-2 motor KIF17 is regulated by importin-beta2 and RanGTP. Nat. Cell Biol..

[48] Follit J.A., Li L., Vucica Y., Pazour G.J. (2010). The cytoplasmic tail of fibrocystin contains a ciliary targeting sequence. J. Cell Biol..

[49] Tao B., Bu S., Yang Z., Siroky B., Kappes J.C., Kispert A., Guay-Woodford L.M. (2009). Cystin localizes to primary cilia via membrane microdomains and a targeting motif. J. Am. Soc. Nephrol..

[50] Barbeito P., Tachibana Y., Martin-Morales R., Moreno P., Mykytyn K., Kobayashi T., Garcia-Gonzalo F.R. (2021). HTR6 and SSTR3 ciliary targeting relies on both IC3 loops and C-terminal tails. Life Sci. Alliance.

[51] Schou K.B., Pedersen L.B., Christensen S.T. (2015). Ins and outs of GPCR signaling in primary cilia. EMBO Rep..

[52] Chen X., Zaro J.L., Shen W.-C. (2013). Fusion protein linkers: Property, design and functionality. Adv. Drug Deliv. Rev..

[53] Snapp E. (2005). Design and use of fluorescent fusion proteins in cell biology. Curr. Protoc. Cell Biol..

[54] Bindels D.S., Haarbosch L., van Weeren L., Postma M., Wiese K.E., Mastop M., Aumonier S., Gotthard G., Royant A., Hink M.A. (2017). mScarlet: A bright monomeric red fluorescent protein for cellular imaging. Nat. Methods.

[55] Los G.V., Encell L.P., McDougall M.G., Hartzell D.D., Karassina N., Zimprich C., Wood M.G., Learish R., Ohana R.F., Urh M. (2008). HaloTag: A novel protein labeling technology for cell imaging and protein analysis. ACS Chem. Biol..

[56] Sharpe H.J., Stevens T.J., Munro S. (2010). A comprehensive comparison of transmembrane domains reveals organelle-specific properties. Cell.

[57] Munro S. (1995). A comparison of the transmembrane domains of Golgi and plasma membrane proteins. Biochem. Soc. Trans..

[58] Hung V., Udeshi N.D., Lam S.S., Loh K.H., Cox K.J., Pedram K., Carr S.A., Ting A.Y. (2016). Spatially resolved proteomic mapping in living cells with the engineered peroxidase APEX2. Nat. Protoc..

[59] May E.A., Kalocsay M., D’Auriac I.G., Schuster P.S., Gygi S.P., Nachury M.V., Mick D.U. (2021). Time-resolved proteomics profiling of the ciliary Hedgehog response. J. Cell Biol..

[60] Hockemeyer D., Soldner F., Beard C., Gao Q., Mitalipova M., DeKelver R.C., Katibah G., Amora R., A Boydston E., Zeitler B. (2009). Efficient targeting of expressed and silent genes in human ESCs and iPSCs using zinc-finger nucleases. Nat. Biotechnol..

[61] Guadiana S.M., Semple-Rowland S., Daroszewski D., Madorsky I., Breunig J.J., Mykytyn K., Sarkisian M.R. (2013). Arborization of dendrites by developing neocortical neurons is dependent on primary cilia and type 3 adenylyl cyclase. J. Neurosci..

[62] Macarelli V., Leventea E., Merkle F.T. (2023). Regulation of the length of neuronal primary cilia and its potential effects on signalling. Trends Cell Biol..

[63] Miyoshi K., Kasahara K., Miyazaki I., Asanuma M. (2009). Lithium treatment elongates primary cilia in the mouse brain and in cultured cells. Biochem. Biophys. Res. Commun..

[64] Hierck B.P., Van der Heiden K., Alkemade F.E., Van de Pas S., Van Thienen J.V., Groenendijk B.C., Bax W.H., Van der Laarse A., DeRuiter M.C., Horrevoets A.J. (2008). Primary cilia sensitize endothelial cells for fluid shear stress. Dev. Dyn..

[65] Rosenbaum J.L., Carlson K. (1969). Cilia regeneration in Tetrahymena and its inhibition by colchicine. J. Cell Biol..

[66] Berbari N.F., Lewis J.S., Bishop G.A., Askwith C.C., Mykytyn K. (2008). Bardet-Biedl syndrome proteins are required for the localization of G protein-coupled receptors to primary cilia. Proc. Natl. Acad. Sci. USA.

[67] Madugula V., Lu L. (2016). A ternary complex comprising transportin1, Rab8 and the ciliary targeting signal directs proteins to ciliary membranes. J. Cell Sci..

[68] Geneva I.I., Tan H.Y., Calvert P.D. (2017). Untangling ciliary access and enrichment of two rhodopsin-like receptors using quantitative fluorescence microscopy reveals cell-specific sorting pathways. Mol. Biol. Cell.

[69] Zhang L., Leng Q., Mixson A.J. (2005). Alteration in the IL-2 signal peptide affects secretion of proteins in vitro and in vivo. J. Gene Med..

