# Supplementary material for: A Short Sequence Targets Transmembrane Proteins to Primary Cilia
Source: Cells. 2024 Jul 6;13(13):1156. doi: 10.3390/cells13131156 (PMC11240719; doi:10.3390/cells13131156)
Supplement: Supplementary file 1 [file cells-13-01156-s001.zip › Supplementary_Images and Table.pdf]

Key resources table.REAGENT or RESOURCE	SOURCE	CATALOG
**Bacterial strains**		
E. coli HST08	Takara Bio	636763
**Chemicals, peptides, and recombinant proteins**		
Synthemax II-SC Substrate	Sigma-Aldrich	CLS3535-1EA
Geltrex Matrix	ThermoFisher Scientific	A1413202
Poly-L-ornithine hydrobromide	Sigma-Aldrich	P3655
Laminin 511	Takara bio	T304
DMEM-F12 Medium	Sigma-Aldrich	D6421
DMEM Medium	Corning	10-013-CM
DMEM/F12 with HEPES Medium	ThermoFisher Scientific	11-330-032
DMEM/F12 with GlutaMAX	ThermoFisher Scientific	31331093
StemFlex Medium	ThermoFisher Scientific Scientific	A3349401
BrainPhys Neuronal Medium	STEMCELL Technologies	05790
Neurobasal- A	ThermoFisher Scientific	10888022
Opti-MEM I Reduced Serum Medium	ThermoFisher Scientific	31985070
Tryple-Express	Life Technologies	12604021
Trypsin-EDTA	Sigma-Aldrich	T3924
Ethylenediaminetetraacetic acid (EDTA)	Sigma-Aldrich	03690
Papain	Worthington Biochemical	LS003120
Dnase	Worthington Biochemical	LK003170
L-Glutamine	Sigma-Aldrich	G7513
FBS	Gibco	10270106
Pen-Strep	Sigma-Aldrich	P0781
Glutamax	ThermoFisher Scientific	35050038
Sodium Bicarbonate	Thermo Fisher Scientific	25080-094
Ascorbic acid	Sigma Aldrich	A4403
MEM Non-Essential amino acids	ThermoFisher Scientific	11140035
N-2 Supplement	ThermoFisher Scientific	17502048
B27 supplement	ThermoFisher Scientific	17504001
Rock Inhibitor Y-27632	DNSK International	DNSKKI-15-02
Blasticidin S HCl	Thermo-Fisher Scientific	R21001
Puromycin Dihydrochloride	ThermoFisher Scientific	A1113803
Doxycycline hydrochloride	Sigma-Aldrich	D3447
2-Mercaptoethanol	Thermo-Fisher Scientific	31350010
Uridine	Scientific Lab	U3003
5-Fluoro-2’-deoxyuridine	Sigma Aldrich	F0503
1 µM Alt-R™ HDR	IDT	10007910
Human GDNF	PeproTech	450-10
Human BDNF	Qkine	Qk050
Human NT-3	Cambridge Bioscience	GFH99

Laminin	Sigma Aldrich	L2020
PD0332991 isethionate	Sigma Aldrich	PZ0199
DAPT	DNSK International	DNSK-EI-01
Calcium chloride solution	Merck	21115
LM22A4	Tocris	4607/5
CHIR99021	Cambridge Bioscience	SM13
GABA	Tocris	0344
NKH 447	Sigma Aldrich	N3290
Lithium chloride	Sigma Aldrich	L9650
Colchicine	Sigma Aldrich	C9754
Lipofectamine™ LTX Reagent with PLUS™ Reagent	ThermoFisher Scientific	15338030
HaloTag Alexa Fluor 488 Ligand	Promega	G1002
JF503 HaloTag Ligand	Janelia Materials	n/a
JF635i HaloTag Ligand	Janelia Materials	n/a
16% Formaldehyde	ThermoFisher Scientific	28908
Nromal Donkey serum	Stratech Scientific	017-000-121-JIR
DAPI (4′,6-diamidino-2-phenylindole)	ThermoFisher Scientific	D1306
PhenoPlateTM 96-well microplates	Perkin Elmer	6055302
**Commercial kits**		
In-Fusion HD EcoDry Cloning Plus Kit	Takara Bio	638913
pENTR™/D-TOPO™ Cloning Kit .	ThermoFisher Scientific	K240020
Gateway™ LR Clonase™ II enzyme reaction	Invitrogen	11791020
P3 Primary Cell 4D-Nucleofector kit	Lonza	V4XP-3024
**Experimental models: Cell lines**		
mIMCD3	Mouse epithelial (kidney)	RRID:CVCL_0429
NIH-3T3	Mouse fibroblast	RRID:CVCL_0594
hTERT RPE-1	Human retinal pigment epithelial	RRID:CVCL_4388
WTC11	Human induced pluripotent stem cell line	RRID:CVCL_Y803
KOLF2.1J	Human induced pluripotent stem cell line	RRID:CVCL_B5P3
**Oligonucleotides**		
AAVS1-5′-F-3 for genomic PCR	AGCTCCCATAGCTCA GTCTGG	n/a
PolyA-R-3 for genomic PCR	GCACCTTCCAGGGTC AAGGAA	n/a
PURO-R-3 for genomic PCR	CGTGGGCTTGTACTC GGTCAT	n/a
TK-F-1 for genomic PCR	TACCTCCGGGATGAT CCAGAC	n/a
AMP-R-1 for genomic PCR	GGTGAGCAAAAACA GGAAGGC	n/a

APEX2-F for PCR cloning	CCGGGATCCACCGGTCGGTTCAGGAAAGTCTTACCCAACTGTGAGT	n/a
APEX2 -R or PCR cloning	CATGGTGGCGACCGGGCTGCCACCGCCGCCGGCATCAGCAAACCCAAG	n/a
CTS-APEX2-SCARLET-F for PCR cloning	CACCGCTAGCGCCACCATGTAC	n/a
CTS-APEX2-SCARLET-R for PCR cloning	GCTTTACTTGTACAGCTCG	n/a
**Recombinant DNA**		
pScarlet-N1	Addgene	128060
pEF5B-FRT-cilia-APEX plasmid	Addgene	73186
pENTR™ TOPO® plasmid	ThermoFisher Scientific	K240020
pAAVS1-CAG-DEST-DTA-TK	[36]	n/a
pAAVS1-P-CAG-DEST	Addgene	RRID:Addgene_80490
pDTA-TK	Addgene	RRID:Addgene_22677
**Software and algorithms**		
HaloTag+ cilia Harmony pipeline	Available upon request	n/a

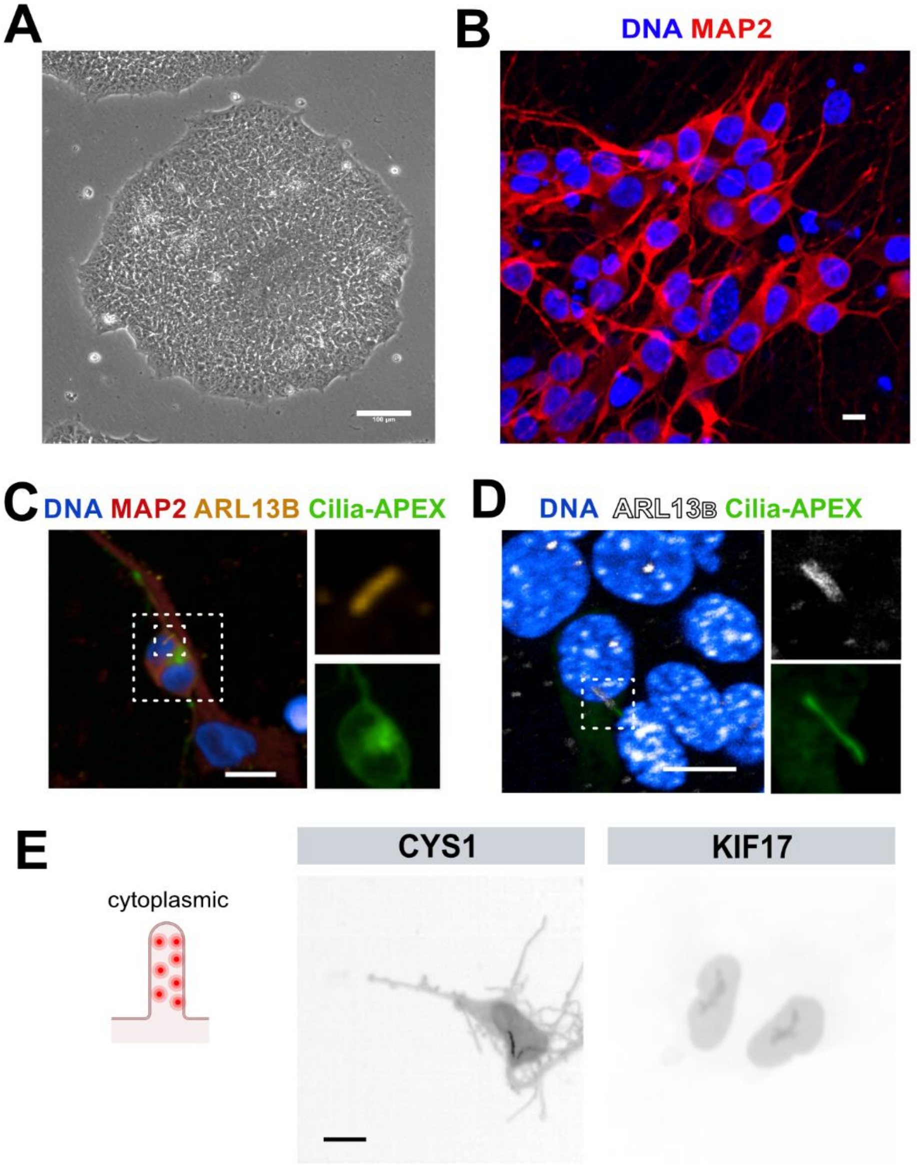

Figure S1. Neuronal differentiation of hiPSCs and testing of ciliary localisation. (A) Representative image of a colony of KOLF2.1J hiPSCs. **(B)** Human iPSC-derived neurons immunostained for the marker MAP2. (**C,D)** Upon transient transfection of Cilia-APEX into neurons, GFP signal is seen throughout the cell but not selectively localized to primary cilia (**C**), but the construct localizes exclusively to cilia in IMCD3 ciliated cell line (**D**). (**E)** Cells transfected with cytoplasmic constructs with CTSs derived from CYS1 and KIF17 do not localize to neuronal primary cilia. Scale bars represent 100 μm for A and 10 μm B-E.

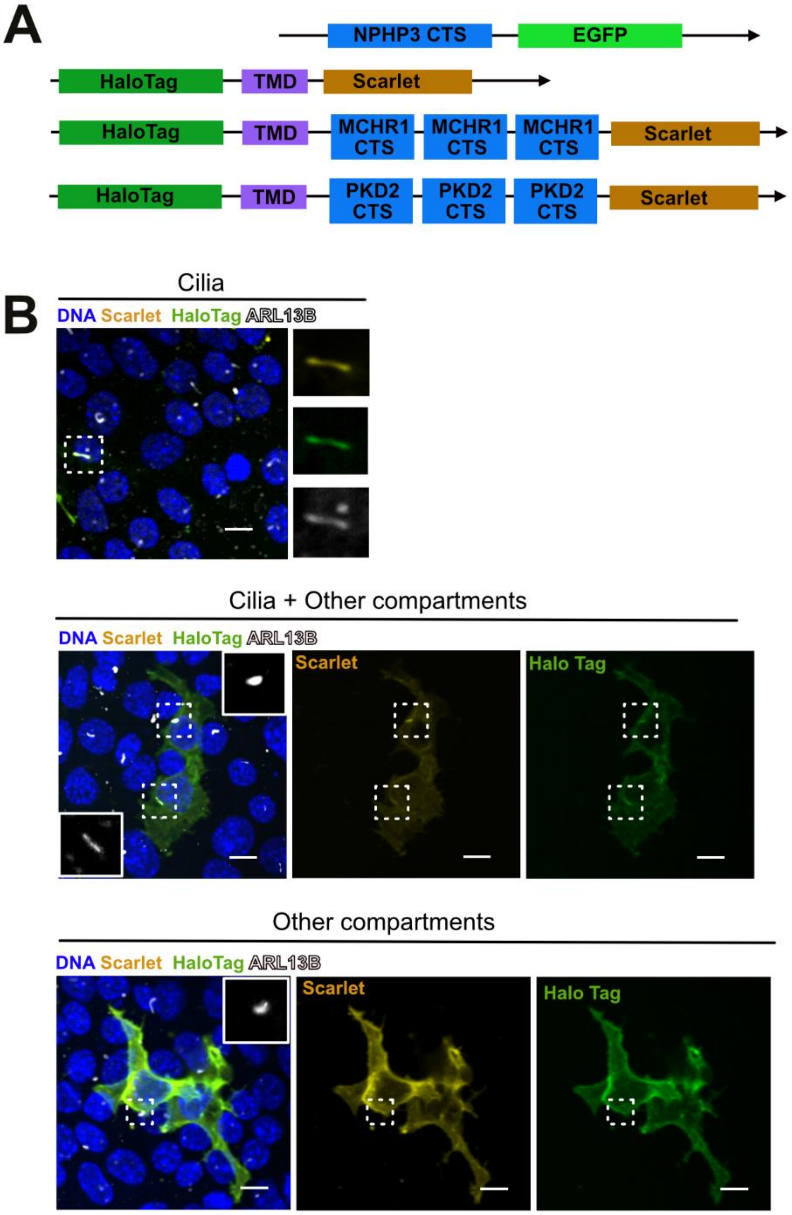

Figure S2. Cilia targeting constructs design and their localisation in diverse ciliated cell lines. (A) Schematic design of the Cilia-APEX construct (top), TM control construct lacking ciliary localization sequences, and constructs with CTSs from MCHR1 and PKD2. (**B)** Representative images of cells with exclusively ciliary localisation, mixed ciliary and non-ciliary localisation, or exclusively non-ciliary localisation. Scale bars represent 10 μm.

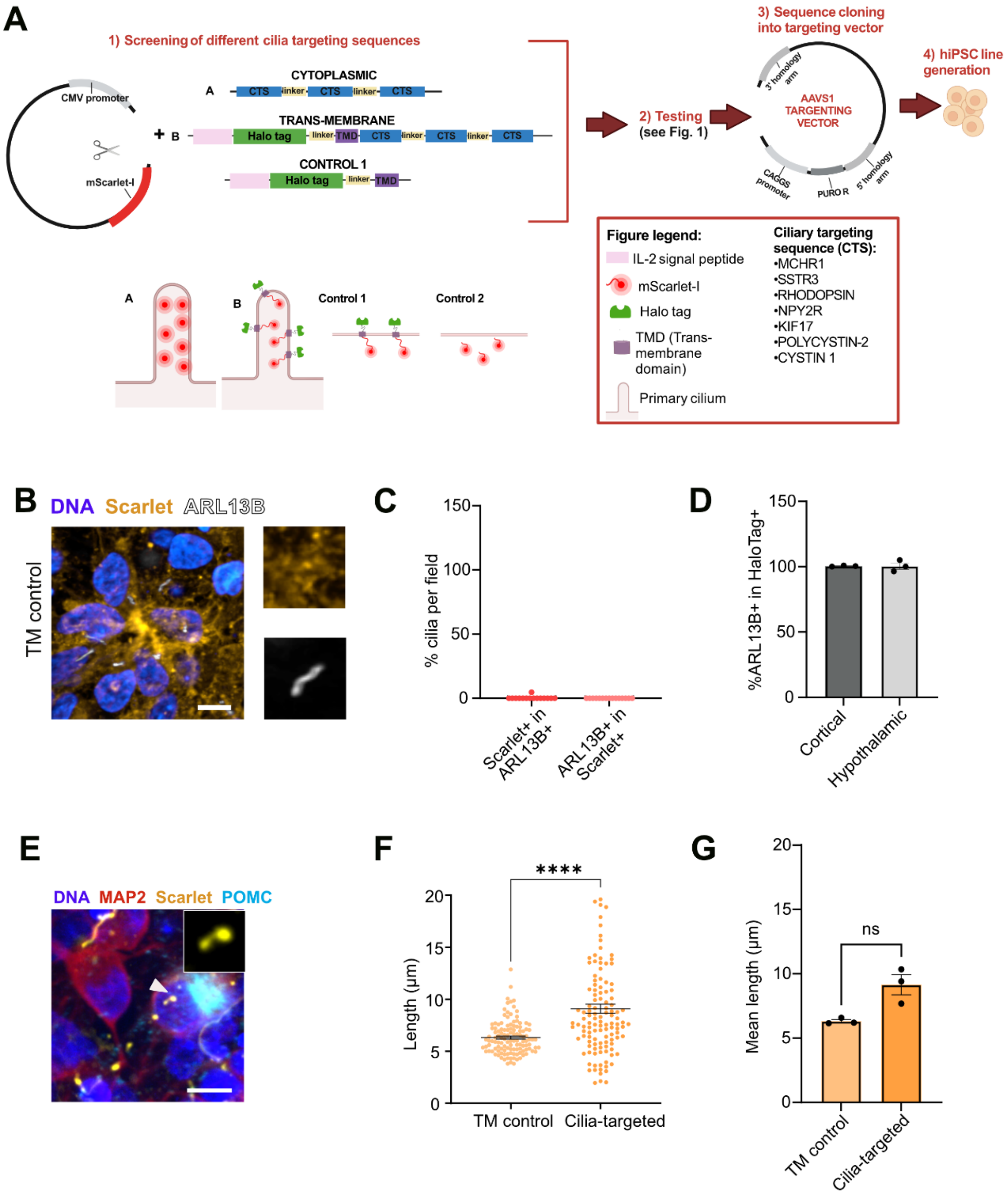

Figure S3. Design and testing of ciliary reporter cell lines. (A) Experimental schematic for 1) screening different cilia targeting sequences by transient transfection of expression plasmids driven by a CMV promoter and visualized by mScarlet, followed by 2) testing by transient transfection, 3) cloning sequences into an AAVS1 targeting vector with positive and negative selection, and 4) generation of stable hiPSC lines. (**B)** The stable hiPSC line carrying the TM control plasmid that lacks CTSs does not show mScarlet co-localisation with ARL13B. **C)** Quantification of ARL13B+ structures expressing mScarlet in cells stably expressing the TM control construct, n = 311 ARL13B+ structures. (**D)** Quantification of HaloTag-expressing structures that co-express ARL13B in hiPSC-derived cortical and hypothalamic cells. N = 3 technical replicates, n = 25 +/- 3 (hypothalamic) and 266 +/- 55 (cortical) HaloTag+ structures per replicate. (**E)** Human iPSC-derived hypothalamic neurons immunopositive for pro-opiomelanocortin (POMC) have primary cilia

that are labeled by endogenous mScarlet fluorescence. Scale bars represent 10 μm in B and E. (**F,G**) Quantification of ARL13B+ structures in human iPSC-derived hypothalamic neurons expressing either the TM control construct or the MCHR1-3xCTS primary cilia reporter construct (Cilia-targeted). N = 3 technical replicates, 30 fields/replicate, and 40 quantified structures per replicate. Each data point represents one primary cilium in F and one technical replicate in G. ****$p<0.0001$ by Mann Whitney test. Bar indicates mean cilia length + SEM.

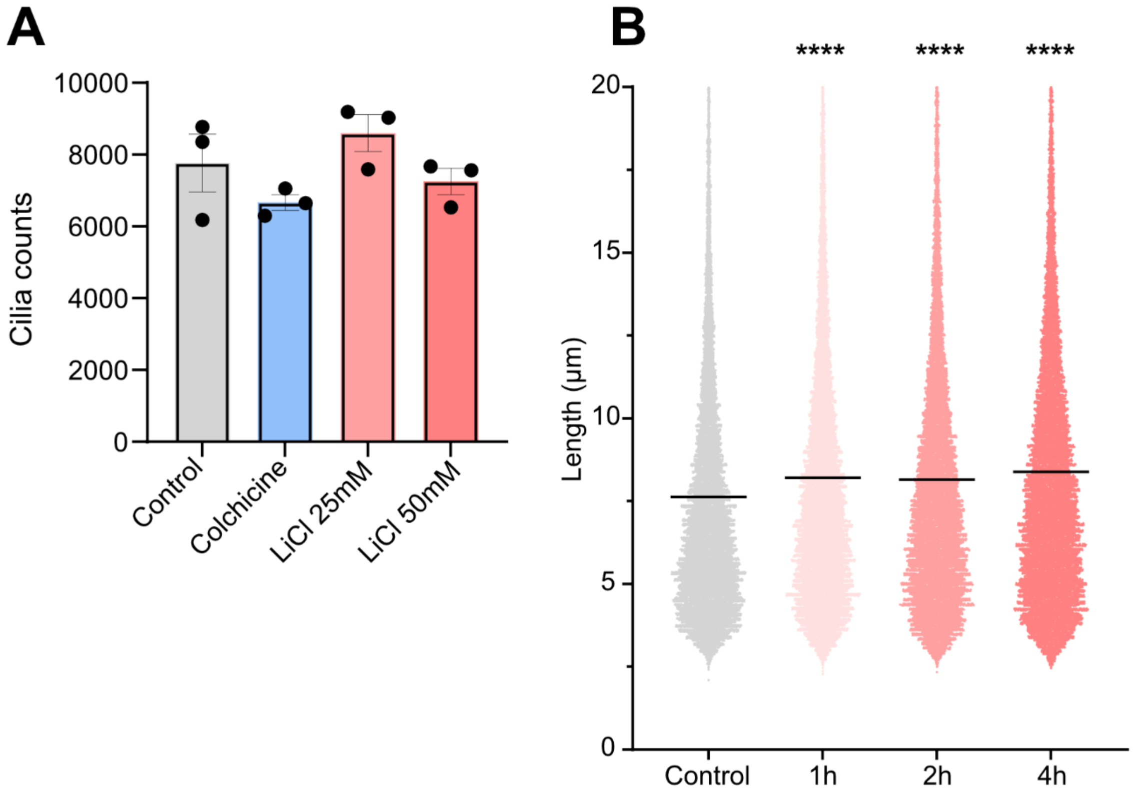

Figure S4. Effects of LiCl and colchicine on primary cilia length in human neurons. (A) Total cilia numbers quantified across parallel cultures of hiPSC-derived hypothalamic neurons and treated with colchicine or LiCl are similar. (**B**) Neuronal cultures treated with a vehicle control or 25 mM LiCl for 1, 2, or 4 hours show time-dependent changes in primary cilia length relative to controls. N = 3 technical replicates per condition, and 8328 +/- 720 quantified structures per replicate. Bar indicates mean cilia length. ****p<0.0001 by Kruskal-Wallis test.
