# Supplementary figures and images for: A Short Sequence Targets Transmembrane Proteins to Primary Cilia
Source: Cells. 2024 Jul 6;13(13):1156. doi: 10.3390/cells13131156 (PMC11240719; doi:10.3390/cells13131156)

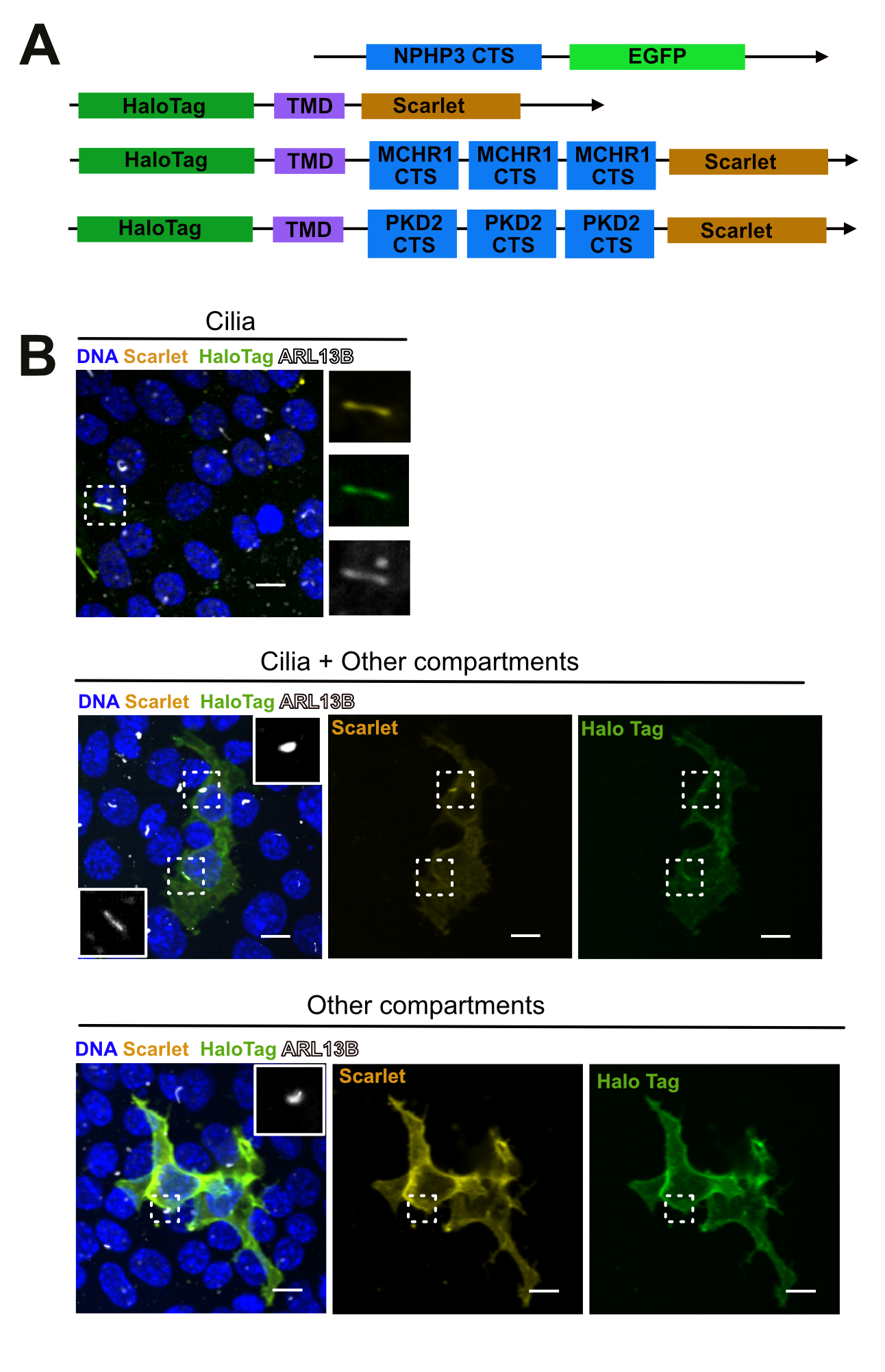

Supplement: Supplementary file 1 [file cells-13-01156-s001.zip › Figure S2_v9_04.07.24.jpg]

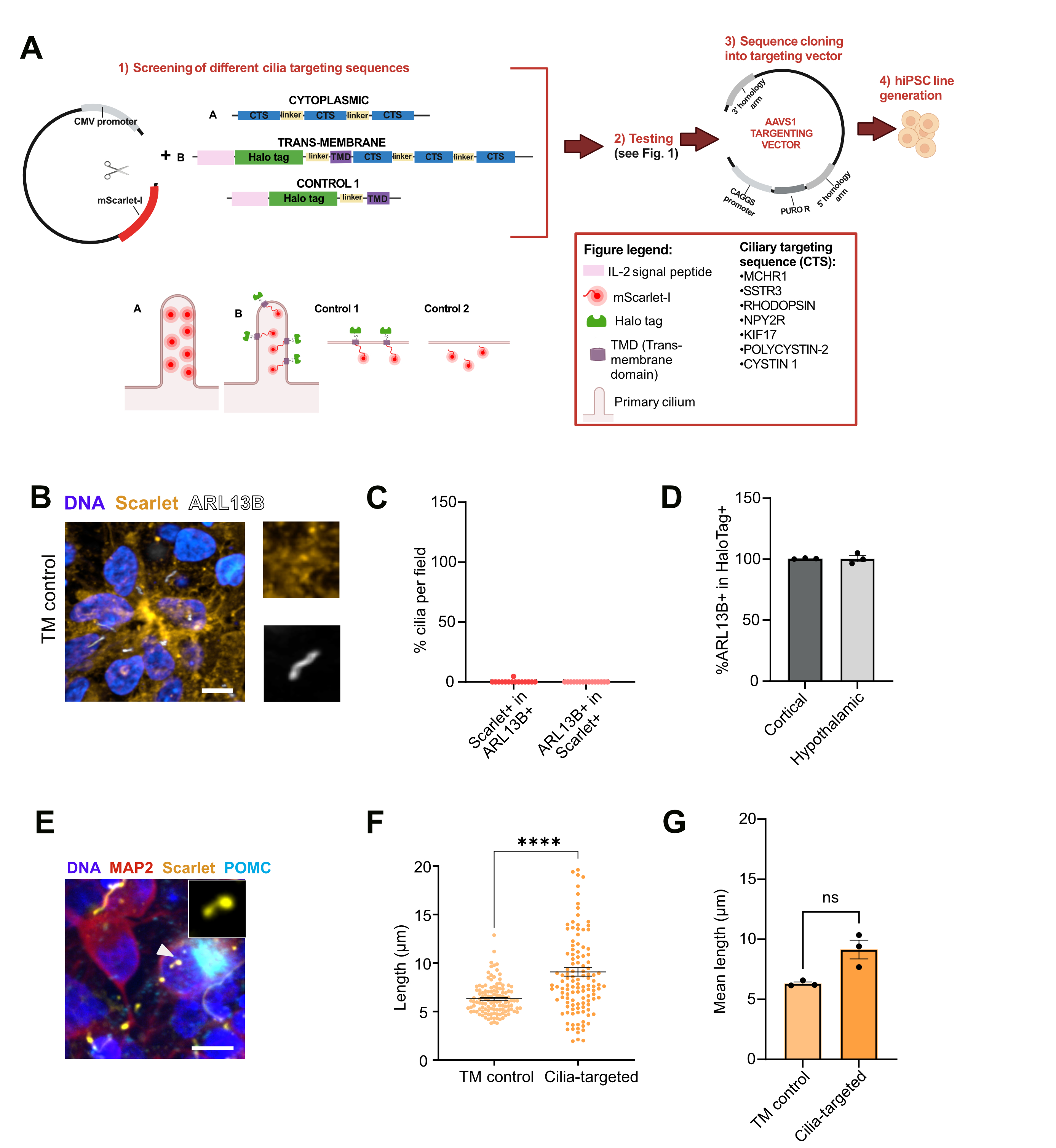

Supplement: Supplementary file 1 [file cells-13-01156-s001.zip › Figure S3_v8_04.07.24.jpg]

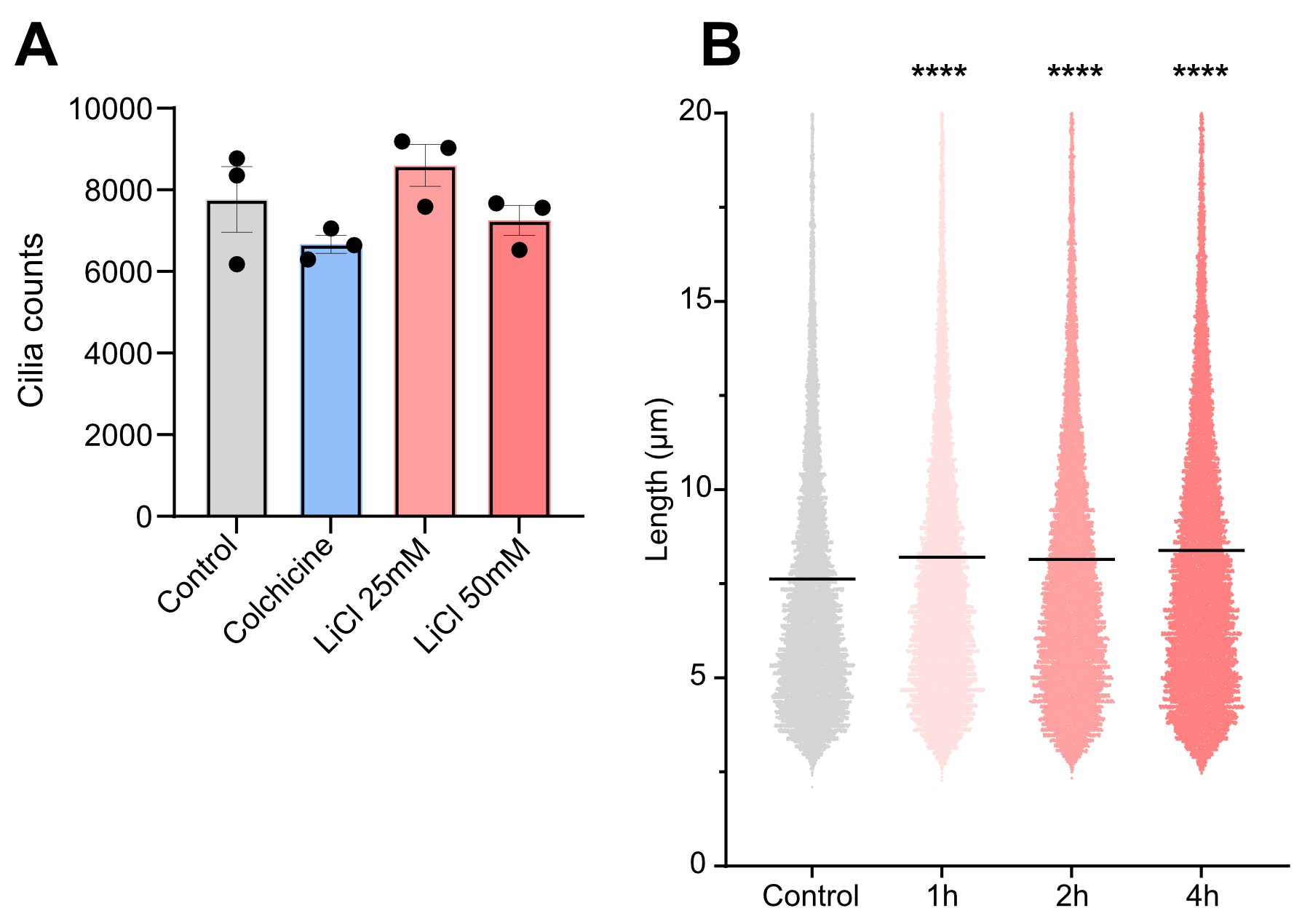

Supplement: Supplementary file 1 [file cells-13-01156-s001.zip › Figure S4_v6_24.06.24.jpg]

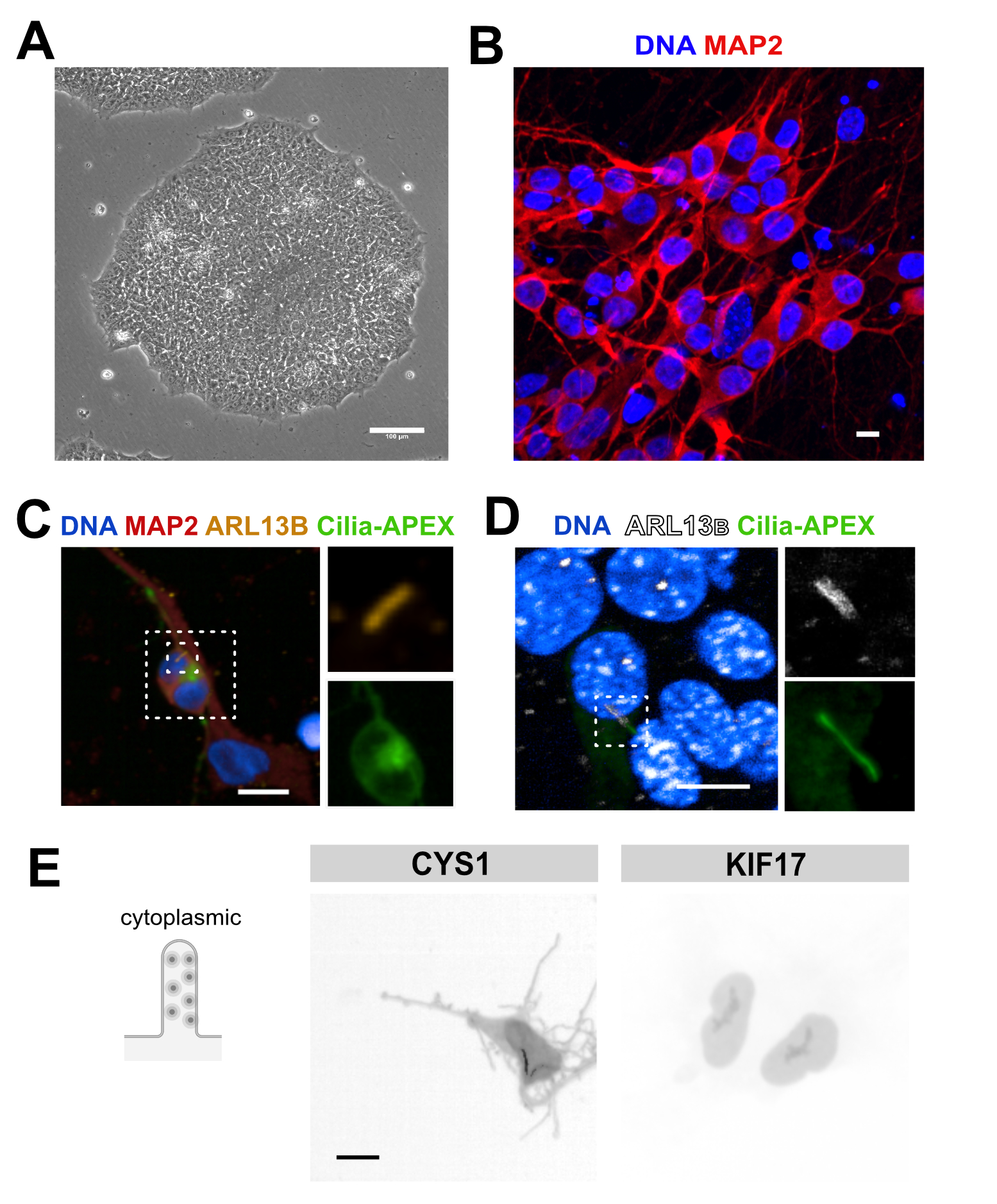

Supplement: Supplementary file 1 [file cells-13-01156-s001.zip › FigureS1_v9_29.03.24.jpg]
